# Genomic characterization of ESBL-producing *Escherichia coli* isolates from patients in an orthopaedic ward in Mwanza, Tanzania

**DOI:** 10.12688/f1000research.180294.1

**Published:** 2026-05-05

**Authors:** Gerald Mboowa, Benson R Kidenya, Ivan Sserwadda, Stephen Kanyerezi, Jeremiah Seni

**Affiliations:** 1 Department of Immunology and Molecular Biology, College of Health Sciences, School of Biomedical Sciences, Makerere University, Kampala, P.O. Box 7072, Uganda; 2 Weill Bugando School of Medicine, Catholic University of Health and Allied Sciences, Mwanza, P.O. Box 1464, Tanzania

**Keywords:** Escherichia coli, ESBL (extended-spectrum β-lactamase), antimicrobial resistance, hospital environment, whole-genome sequencing

## Abstract

**Background:**

Extended-spectrum β-lactamase (ESBL)-producing *Escherichia coli* are a major contributor to antimicrobial resistance (AMR) in healthcare settings, yet their genomic diversity and population structure remain poorly characterized in low-resource settings. Orthopaedic wards may facilitate persistence and transmission due to prolonged hospitalization, frequent antimicrobial exposure, and increased patient vulnerability. This study investigated the genomic characteristics of ESBL-producing *E. coli* recovered from hospitalized patients in an orthopaedic ward in Mwanza, Tanzania.

**Methods:**

We performed whole-genome sequencing and comparative genomic analysis of ESBL-confirmed *E. coli* isolates obtained from stool or rectal swabs of hospitalized patients. Sequence reads were quality controlled and assembled using standard pipelines. Core genome single nucleotide polymorphism (SNP) alignments were generated using Snippy, and recombination was identified and masked using Gubbins prior to phylogenetic reconstruction. Sequence types (STs), AMR determinants, plasmid replicons, and virulence-associated genes were identified using established bioinformatics tools and databases.

**Results:**

Phylogenetic analysis revealed a polyclonal population structure comprising multiple distinct lineages rather than a single outbreak clone. Several isolates formed tight phylogenetic clusters within the ward, consistent with local circulation of closely related strains. The population included internationally recognized sequence types, including ST131, ST1193, ST69, ST617, and ST38. ESBL production was predominantly associated with *bla*_CTX-M-15_, detected in most isolates, alongside additional resistance determinants to aminoglycosides, quinolones, sulfonamides, trimethoprim, and tetracyclines. IncF-family plasmid replicons predominated, with additional IncY plasmids identified. Virulence-associated genes, including *fdeC* and *ybtP/ybtQ*, were widely distributed.

**Conclusions:**

ESBL-producing *E. coli* in this orthopaedic ward represent a genomically diverse, multidrug-resistant population dominated by *bla*_CTX-M-15_-positive lineages. The coexistence of multiple lineages alongside closely related clusters suggests ongoing circulation of high-risk clones. These findings highlight colonized patients as reservoirs of AMR and underscore the need to integrate genomic surveillance into infection prevention and control strategies in resource-constrained healthcare settings.

## Introduction

Antimicrobial resistance (AMR) among Gram-negative bacteria represents a major global public health threat, with extended-spectrum *β*-lactamase (ESBL)-producing *Escherichia coli* emerging as one of the most clinically significant pathogens in both community and healthcare settings. ^1, 2^ ESBL enzymes, particularly those of the CTX-M family, confer resistance to third-generation cephalosporins and are frequently co-associated with resistance to other antimicrobial classes, resulting in multidrug-resistant (MDR) phenotypes that complicate treatment and increase morbidity and mortality. ^3– 5^ Among these, *bla*_CTX-M-15_ has become the dominant ESBL gene globally and is widely disseminated across diverse *E. coli* lineages and geographic regions. ^6, 7^

In sub-Saharan Africa, the burden of ESBL-producing Enterobacteriaceae is increasing, driven by limited diagnostic capacity, high antimicrobial usage, and constrained infection prevention and control (IPC) infrastructure. ^8– 10^ *E. coli* is a major contributor to this burden, not only as a cause of invasive infections but also as a colonizer of the gastrointestinal tract, where it serves as a reservoir for AMR genes. ^11, 12^ Colonization is particularly important in hospitalized patients, as it can precede infection and facilitate onward transmission within healthcare settings. ^12– 20^ However, genomic data describing the population structure, resistance determinants, and transmission dynamics of ESBL-producing *E. coli* in African hospital environments remain limited.

Orthopaedic wards represent a high-risk setting for the persistence and dissemination of MDR organisms. ^21– 24^ Patients in these settings often experience prolonged hospital stays, repeated exposure to antibiotics, and increased vulnerability due to underlying trauma or surgical interventions. ^25^ These factors create conditions that may facilitate the maintenance and spread of resistant bacterial populations within the patient cohort. Understanding the diversity and relatedness of colonizing strains in such settings is therefore essential for informing infection prevention and antimicrobial stewardship strategies.

Whole-genome sequencing (WGS) has transformed the study of bacterial epidemiology by enabling high-resolution characterization of population structure, resistance mechanisms, and clonal relationships. ^26^ Genomic approaches allow discrimination between clonal expansion and polyclonal populations, identification of high-risk sequence types such as ST131 and ST1193, and detection of mobile genetic elements, including plasmids that mediate the spread of AMR genes. ^27– 29^ Despite these advances, relatively few studies have applied WGS to investigate ESBL-producing *E. coli* colonization in hospitalized patient populations in sub-Saharan Africa.

Data from Tanzanian healthcare settings are particularly scarce, especially with respect to genomic characterization of ESBL-producing *E. coli* in high-risk hospital wards. In this study, we performed a genomic epidemiological analysis of ESBL-confirmed *E. coli* isolates recovered from stool or rectal swabs of patients admitted to an orthopaedic ward in a tertiary hospital in Mwanza, Tanzania. By integrating WGS with detailed clinical metadata, we aimed to (i) characterize the population structure and sequence types of ESBL-producing *E. coli*, (ii) define their AMR, plasmid, and virulence-associated profiles, and (iii) assess patterns of genomic relatedness consistent with the circulation of multidrug-resistant lineages within the patient population.

## Methods

### Study setting and design

This study was nested within a larger longitudinal investigation conducted over a 4-month period, from 3 January 2020 to 30 May 2020, assessing gastrointestinal colonization with ESBL-producing Enterobacteriaceae among orthopaedic patients admitted to a tertiary hospital in Mwanza, Tanzania. ^30^ The parent study included broader sampling activities within the ward. For this study, we analyzed ESBL-confirmed *E. coli* isolates recovered from stool or rectal swabs collected from patients admitted to the orthopaedic ward. By integrating whole-genome sequencing data with available clinical and ward-level metadata, the study aimed to investigate the population structure of ESBL-producing *E. coli*, define their AMR and associated genomic features, and assess patterns of clustering consistent with the circulation of multidrug-resistant lineages within the orthopaedic ward.

### Bacterial isolation and ESBL confirmation

Clinical stool and rectal swab samples were processed using standard microbiological procedures at the Department of Microbiology and Immunology, Weill Bugando School of Medicine, Catholic University of Health and Allied Sciences, Mwanza, Tanzania. Samples were inoculated onto MacConkey agar (Oxoid, UK) and CHROMagar™ ESBL (CHROMagar, France), a selective medium containing cephalosporins for the isolation of ESBL-producing Gram-negative bacteria. Plates were incubated aerobically at 35–37 °C for 18–24 hours. Colonies with morphology consistent with *E. coli* were selected and subcultured to obtain pure isolates. ESBL production was then confirmed phenotypically using the combination disk method, comparing inhibition zones for cefotaxime and ceftazidime, alone and in combination with clavulanic acid, in accordance with CLSI recommendations for phenotypic ESBL confirmation. ^31^ Only isolates confirmed as ESBL-producing *E. coli* were included in downstream genomic analyses.

### DNA extraction, library preparation, and sequencing

Pure *E. coli* isolates were shipped to the Genomics Laboratory, Department of Immunology and Molecular Biology, College of Health Sciences, Makerere University, Kampala, Uganda, where genomic DNA was extracted using standardized protocols. DNA concentration was measured using the Qubit dsDNA High Sensitivity Assay (Thermo Fisher Scientific, USA), and DNA purity was assessed using a NanoDrop spectrophotometer (Thermo Fisher Scientific, USA) by evaluating A260/280 and A260/230 ratios. DNA integrity was further assessed by 1.0% agarose gel electrophoresis. Whole-genome sequencing was performed at the Earlham Institute (Norwich, UK) using the Illumina NovaSeq 6000 platform. Sequencing libraries were prepared using the LITE (Low Input Transposase-Enabled) protocol, and sequencing was carried out using paired-end 150-bp chemistry (2 × 150 bp reads).

### Read processing and genome assembly

Raw sequencing reads were subjected to quality control using FastQC (v0.12.1), ^32^ followed by adapter trimming and removal of low-quality bases using Trimmomatic (v0.40), ^33^ with default parameters. High-quality reads were retained for downstream analyses. *De novo* genome assemblies were generated using SKESA (Strategic *K*-mer Extension for Scrupulous Assemblies) assembler (v2.4.0), ^34^ with default settings and assembly quality was assessed using standard metrics, including genome size, contiguity, and N50 values, to ensure suitability for comparative genomic analysis.

### Genomic species confirmation

Genomic species confirmation was performed using a multi-tool approach to ensure robust and accurate taxonomic classification. Draft genome assemblies were analyzed using GTDB-Tk (v2.6.0) ^35^ (Genome Taxonomy Database Toolkit), which assigns taxonomy based on genome-wide phylogenetic placement against the GTDB reference database.

To complement this, GAMBIT v1.1.0 (Genomic Approximation Method for Bacterial Identification and Tracking) ^36^ was used to provide *k*-mer–based taxonomic classification, enabling rapid and high-resolution identification of bacterial genomes. In addition, Basic Local Alignment Search Tool for nucleotides (BLASTn) searches were performed against the NCBI nucleotide database to confirm species identity based on sequence similarity to well-characterized reference genomes. Blast analyses were conducted using the Docker image gmboowa/blast-analysis:1.9.4.

Concordance across these three independent approaches (GTDB-Tk, GAMBIT, and BLAST) was used to validate species assignments, ensuring all isolates were accurately classified as *E. coli* prior to downstream comparative genomic analyses.

### Genome characterization and typing

Sequence types (STs) were assigned from draft genome assemblies using the MLST module in rMAP-2.0, implemented with the mlst tool (Torsten Seemann; Docker image staphb/mlst:2.19.0). This approach scans assembled contigs against PubMLST-curated schemes to determine the sequence type and allele profile for each isolate, enabling classification into globally recognized *E. coli* lineages. ^37^

### Read mapping and variant calling

Quality-controlled reads were mapped to the clinical *E. coli* reference genome GCF_000285655.3_EC958.v1 using the Snippy pipeline (v4.6.0), ^38^ which integrates BWA-MEM for read alignment and bcftools for variant calling. This clinically relevant reference was selected to improve mapping accuracy and reduce reference bias in the analysis of hospital-associated isolates. Core genome single nucleotide polymorphisms (SNPs) were identified across all isolates, and a multiple sequence alignment of core SNPs was generated for downstream phylogenetic analysis.

### Recombination filtering and phylogenetic reconstruction

To account for homologous recombination, SNP alignments were processed using Gubbins v3.4.1 (Genealogies Unbiased By recomBinations In Nucleotide Sequences), ^39^ which identifies and masks regions of elevated SNP density consistent with recombination. The resulting recombination-filtered alignment represents clonal variation across the dataset. Maximum-likelihood phylogenetic trees were inferred using IQ-TREE v3.1.1, ^40^ based on the recombination-filtered core genome SNP alignment. Branch support was assessed using standard bootstrap approaches. Pairwise SNP distances between isolates were calculated using snp-dists, providing a quantitative measure of genomic relatedness. The final phylogenetic tree was visualized in iTOL v7.0 (Interactive Tree of Life), ^41^ where isolate metadata were incorporated to annotate the tree and facilitate interpretation of clustering patterns, epidemiological relationships, and potential transmission events.

### Transmission network analysis

To investigate potential transmission events among ESBL-producing *E. coli* isolates, pairwise SNP distances were calculated from the recombination-filtered core genome alignment generated using Snippy and processed with Gubbins. SNP distances were computed using snp-dists, providing a matrix of pairwise genomic distances among the 39 clinical isolates and the included reference genome. A transmission-linked network was constructed using a predefined SNP threshold of ≤5 SNPs, consistent with thresholds commonly applied to infer recent transmission or shared-source acquisition in bacterial genomic epidemiology. Isolates and the reference genome were represented as nodes, and edges were drawn between nodes with pairwise SNP distances at or below this threshold. Edge labels indicate the number of SNP differences between connected nodes. The network was generated using Python (version 3.14) with the NetworkX library for graph construction and Matplotlib for visualization. A force-directed layout algorithm (spring layout) was applied to position nodes based on their connectivity, enabling clear visualization of clusters of closely related isolates and facilitating identification of putative transmission events within the orthopaedic ward.

### Antimicrobial resistance, plasmid, and virulence analysis

Antimicrobial resistance genes were identified using the ResFinder v4.5.0 database, ^42^ enabling classification of resistance determinants into antimicrobial classes. MDR was defined as the presence of resistance determinants spanning three or more antimicrobial classes. ^43^ Plasmid replicons were identified within rMAP-2.0 using PlasmidFinder ^44^ to screen assembled genomes for known plasmids, while virulence-associated genes were detected using ABRicate, ^45^ implemented through the Docker image staphb/abricate:1.0.0 against curated *E. coli* virulence factor databases. These analyses were executed through the containerized rMAP-2.0 workflow, ensuring reproducibility, portability, and consistent processing across all samples. ^37^

### Data integration and comparative analysis

Genomic data were integrated with available epidemiological and clinical metadata, including non-identifiable clinical and epidemiological metadata. This enabled an integrated analysis of population structure, antimicrobial resistance burden, plasmid content, and virulence-associated profiles. Phylogenetic clustering was interpreted in the context of ward-level and patient-level metadata to assess patterns consistent with the circulation and local persistence of closely related multidrug-resistant lineages within the orthopaedic ward.

## Results

### Sample characteristics

A total of 45 ESBL-producing clinical *E. coli* isolates were initially collected from patient-derived stool and rectal swab samples obtained from individuals admitted to the orthopaedic ward. Of these, 39 isolates passed quality control and were included in the whole-genome sequencing and downstream analyses ( Table 1). Core genome phylogenetic analysis revealed multiple distinct clusters, indicating genomic diversity among isolates circulating within the ward. Several isolates formed tight phylogenetic clusters, suggestive of potential shared sources or localized transmission events, while others appeared more genetically distinct. Notably, isolate A55728 formed a long independent branch, indicating substantial divergence from other isolates. The distribution of isolates across phylogenetic clusters, together with metadata on sample source and collection site, provided a framework for investigating potential transmission dynamics between patients and the hospital environment. Detailed isolate-level characteristics are presented in **Extended data 1 (Table S1)**.

**Table 1.** Summary of sample characteristics of 39 ESBL-producing *E. coli* isolates.

Characteristic	Value
Total isolates	39
Source	Patient and environment
Median age (years)	30.5 years
Sex distribution	21 female (53.8%), 18 male (46.2%)
Common fracture types	Closed (66.7%), Open (23.1%)
Most common fracture sites	Femur (38.5%), Tibia (10.3%)
Median genome size (Mb)	~5.1
Median contigs	90 (range: 50–3339)
Median GC (%)	~50.6
MDR isolates	39/39 (100%)
Dominant sequence types (MLST)	ST131, ST1193, ST69
Number of phylogenetic clusters	8 clusters + 1 outlier

### Genomic features

Whole-genome sequencing of 39 ESBL-producing *E. coli* isolates generated high-quality draft assemblies suitable for downstream comparative genomic analysis. Species identity was genomically confirmed using a combination of GTDB-Tk, GAMBIT, **Extended data 1 (Table S1)** and BLAST-based approaches ( https://gmboowa.github.io/rMAP-2.0/reports/esbl_ecoli.html#blast), ensuring accurate taxonomic assignment and minimizing misclassification within the *E. coli* species complex.

The assemblies had a median genome size of approximately 5.1 Mb, consistent with typical *E. coli* genomes. Assembly fragmentation varied across isolates, with a median of 90 contigs (range: 50–3339), indicating overall good assembly quality, although a small number of genomes were highly fragmented. The GC content was highly conserved across isolates, with a median of approximately 50.6%, reflecting the expected genomic composition of *E. coli.*

Sequence typing revealed a genetically diverse population comprising several globally recognized high-risk and community-associated lineages, including ST131, ST1193, ST69, ST617, and ST38, alongside additional less frequent sequence types. This diversity indicates the co-circulation of internationally disseminated high-risk clones and locally established lineages within the orthopaedic ward population.

Core genome SNP analysis using Snippy identified substantial genomic diversity across the isolates. The reference-based alignment revealed a wide range of variant sites, highlighting the presence of both closely related and highly divergent isolates within the same clinical setting. The distribution of SNPs per isolate demonstrated marked heterogeneity, with some isolates showing minimal divergence consistent with recent transmission or shared ancestry, while others exhibited extensive polymorphism, reflecting the presence of multiple independent lineages.

### Antimicrobial resistance, plasmids, and virulence determinants

All 39 isolates were classified as MDR, carrying resistance determinants to three or more antimicrobial classes. ESBL production was predominantly associated with *bla*_CTX-M-15_, which was detected in most isolates and is consistent with its recognized role as a globally disseminated ESBL determinant.

Beyond *β*-lactam resistance, the isolates harbored genes associated with resistance to several additional antimicrobial classes, including aminoglycosides, fluoroquinolones, sulfonamides, trimethoprim, and tetracyclines. This broad resistome highlights the accumulation of multiple resistance determinants within individual isolates and reflects the complex AMR burden present in the orthopaedic ward setting.

Plasmid analysis showed a predominance of IncF-family replicons, which are frequently implicated in the dissemination of ESBL genes in *E. coli.* Additional plasmid types, including IncY, were also identified in a subset of isolates, indicating the involvement of diverse mobile genetic elements in the acquisition and spread of resistance determinants.

Virulence-associated genes were also widely distributed across the isolate collection, including factors linked to adhesion, iron acquisition, and extraintestinal survival, such as *fdeC* and *ybtP/ybtQ.* The co-occurrence of antimicrobial resistance and virulence-associated determinants underscores the clinical relevance of these isolates and raises concern about the circulation of lineages with both enhanced pathogenic potential and limited treatment options.

### Pairwise SNP distances and transmission inference

Pairwise SNP distances were calculated from the recombination-filtered core genome alignment to assess genomic relatedness among *E. coli* clinical isolates. SNP distances ranged widely across the dataset, from 0 to 20,176 SNPs, with a mean distance of approximately 2,506 SNPs, indicating substantial genetic diversity within the orthopaedic ward population. Despite this overall diversity, a subset of isolates exhibited very low SNP distances (0–5 SNPs), consistent with recent transmission events or a shared source within the hospital environment. ^46^ In contrast, the majority of isolate pairs showed large SNP distances (>1000 SNPs), suggesting the presence of genetically unrelated strains and multiple independent introductions into the hospital ward.

### Identification of genetically indistinguishable isolates (0 SNP cluster)

A cluster of isolates with zero SNP differences was identified, comprising isolates A55724, A55793, and A55798. These isolates were genetically indistinguishable at the core genome level ( Table 2), strongly indicating a recent transmission event or a common source. Such zero-distance clustering provides high-confidence evidence of direct or near-direct transmission within the clinical setting.

**Table 2.** Pairwise SNP distances among isolates with zero SNP differences (Cluster 1). This table shows isolate pairs with 0 SNP differences based on core genome SNP analysis, indicating genetically indistinguishable genomes. These isolates (A55724, A55793, A55798) likely represent a recent transmission cluster or a shared source within the orthopaedic ward. Cluster 1 (0 SNP differences — identical genomes).

Isolate 1	Isolate 2	SNP Distance
A55724	A55793	0
A55724	A55798	0
A55793	A55798	0

### Detection of closely related transmission clusters (≤3 SNPs)

A second cluster of closely related isolates was identified, with pairwise SNP distances ranging from 0 to 3 SNPs. This cluster included isolates A55769, A55939, A55941, and A55966 ( Table 3). The low level of genomic variation within this group is consistent with a recent transmission chain or ongoing circulation of a closely related lineage within the ward.

**Table 3.** Pairwise SNP distances among closely related isolates (Cluster 2). This table presents isolate pairs with 0–3 SNP differences, indicating highly related genomes. The isolates (A55769, A55939, A55941, A55966) form a closely related cluster consistent with recent transmission or an ongoing transmission chain within the ward.

Isolate 1	Isolate 2	SNP distance
A55769	A55939	1
A55769	A55941	1
A55769	A55966	3
A55939	A55941	0
A55939	A55966	2
A55941	A55966	2

### Interpretation of transmission dynamics

Together, these findings indicate a mixed epidemiological scenario within the orthopaedic ward. The presence of both genetically indistinguishable isolates and closely related clusters supports the occurrence of localized transmission events, while the broader distribution of high SNP distances across the dataset reflects a diverse background population structure, likely driven by multiple introductions of unrelated *E. coli* lineages into the hospital environment. These results demonstrate the coexistence of recent transmission clusters and genetically diverse strains, underscoring the importance of integrating genomic data with epidemiological context to better understand transmission pathways and inform infection prevention strategies.

Two distinct transmission clusters were identified based on pairwise SNP distances of ≤5 SNPs. The first cluster, comprising isolates A55724, A55793, and A55798, showed 0 SNP differences, indicating genetically indistinguishable isolates and providing strong evidence of recent transmission or a common source. The second cluster, comprising A55769, A55939, A55941, and A55966, showed pairwise SNP distances ranging from 0 to 3 SNPs, consistent with a very closely related group and suggestive of an ongoing transmission chain within the ward.

### Shared variant analysis

Analysis of shared SNPs identified groups of isolates with common variant profiles, further supporting the phylogenetic structure observed in the core genome SNP analysis. Isolates that were closely related in the phylogeny shared a higher proportion of SNPs, forming distinct genomic clusters consistent with possible transmission chains or recent common ancestry. In contrast, isolates with few or no shared variants were more genetically distant, supporting the presence of multiple unrelated lineages within the study population.

### Phylogenetic relationships

Core genome phylogenetic analysis based on the recombination-filtered SNP alignment demonstrated a clearly polyclonal population structure among the 39 ESBL-producing *E. coli* isolates ( Figure 2). The isolates were distributed across multiple distinct lineages, with eight phylogenetic clusters identified alongside one genetically distinct outlier, rather than forming a single outbreak clone. This overall structure indicates substantial genomic diversity within the orthopaedic ward population.

Despite this broad diversity, several isolates formed tight phylogenetic clusters characterized by short branch lengths, consistent with the low pairwise SNP distances observed and suggestive of recent transmission or shared-source exposure within the ward ( Figure 2). In contrast, a subset of isolates displayed long branches, indicating marked genetic divergence and supporting the presence of unrelated strains likely introduced independently into the hospital setting. Notably, isolate A55728 formed a long independent branch and remained clearly separated from the main clusters, consistent with the high SNP distances observed and arguing against its involvement in recent local transmission events.

Taken together, the recombination-filtered phylogeny, pairwise SNP distance analysis, and shared variant patterns support a mixed epidemiological scenario in which localized transmission occurs within a background of multiple independent introductions. The coexistence of closely related isolate clusters and more widely separated lineages suggests that ESBL-producing *E. coli* in this orthopaedic ward are shaped by both local clonal expansion and the repeated introduction of diverse MDR strains. High-risk sequence types, including ST131 and ST1193, were represented across this population, further underscoring their contribution to the dissemination of ESBL-producing *E. coli* in this setting.

### Comparative analysis

Comparative genomic analysis revealed both heterogeneity and shared features across the ESBL-producing *E. coli* population. Although the isolates were distributed across multiple sequence types and phylogenetic clusters, many shared a common repertoire of antimicrobial resistance determinants, most notably *bla*_CTX-M-15_, indicating the widespread distribution of key ESBL-associated resistance genes across genetically distinct lineages.

Comparison of plasmid content further showed that diverse sequence types frequently carried similar IncF-family replicons, supporting a common role for these plasmids in the dissemination of resistance determinants. At the same time, variation in accessory resistance and virulence-associated genes across isolates highlighted the presence of lineage-specific genomic features superimposed on a shared multidrug-resistant background.

Taken together, these findings suggest that the ESBL-producing *E. coli* population in the orthopaedic ward is shaped by both the persistence of successful lineages and the circulation of shared mobile genetic elements, resulting in isolates that are phylogenetically diverse but convergent in their resistance profiles.

## Discussion

This study provides a genomic snapshot of ESBL-producing *E. coli* colonizing patients admitted to an orthopaedic ward in Mwanza, Tanzania, and demonstrates that this patient population harbors a diverse but highly resistant set of lineages. Rather than identifying a single dominant outbreak clone, our data revealed a polyclonal population structure composed of multiple globally recognized and locally circulating sequence types, including ST131, ST1193, ST69, ST617, and ST38. At the same time, the identification of closely related isolate clusters with pairwise distances of 0–3 SNPs indicates that, within this broader diversity, recent transmission or shared-source exposure likely occurred within the ward. Taken together, these findings suggest that ESBL-producing *E. coli* in this setting are maintained through a combination of repeated introduction of diverse strains and localized circulation of specific lineages. ^16, 18^

The predominance of *bla*_CTX-M-15_ across the isolate collection is notable and consistent with its recognized role as the most widely disseminated ESBL determinant in *E. coli.* ^5, 6^ In this study, *bla*_CTX-M-15_ was distributed across multiple phylogenetically distinct backgrounds, indicating that the burden of ESBL production in the ward is not restricted to a single lineage. This pattern supports the view that successful resistance genes can become embedded in diverse strain backgrounds, thereby amplifying their epidemiological and clinical impact. ^3^ The fact that all sequenced isolates were multidrug resistant, with resistance determinants spanning *β*-lactams, aminoglycosides, fluoroquinolones, sulfonamides, trimethoprim, and tetracyclines, further emphasizes the limited therapeutic options associated with these colonizing strains and reinforces the clinical importance of gastrointestinal carriage as a reservoir of difficult-to-treat organisms. ^11, 12^

The plasmid findings provide an important mechanistic explanation for this convergence in resistance profiles across otherwise unrelated lineages. The predominance of IncF-family replicons, together with the detection of additional plasmid types such as IncY, suggests that mobile genetic elements play a major role in shaping the ward resistome. IncF plasmids are particularly important in *E. coli* because of their well-established association with ESBL dissemination and persistence in clinically successful clones. ^28^ In our dataset, the recurrence of similar plasmid backgrounds across multiple sequence types supports the likelihood that plasmid-mediated horizontal gene transfer contributes substantially to the spread of resistance determinants in this setting. Thus, the resistance burden observed here appears to reflect not only the persistence of successful lineages, but also the circulation of shared mobile elements capable of moving across strain backgrounds.

Beyond resistance, the widespread detection of virulence-associated loci, including genes linked to adhesion, iron acquisition, and extraintestinal survival such as *fdeC* and *ybtP/ybtQ*, underscores the clinical relevance of these colonizing isolates. The co-occurrence of antimicrobial resistance and virulence-associated determinants raises concern that the orthopaedic ward patient population may be colonized by strains with both enhanced pathogenic potential and reduced susceptibility to commonly used antibiotics. This is especially important in orthopaedic settings, where prolonged hospitalization, surgical interventions, trauma-related wounds, and repeated antimicrobial exposure may increase opportunities for colonization, persistence, and progression to infection. ^11, 12^ From an infection prevention perspective, colonized patients should therefore be viewed not simply as passive carriers, but as potential reservoirs of clinically important multidrug-resistant organisms. ^21– 24^

The pairwise SNP analysis adds an important epidemiological dimension to these genomic findings. Although the overall SNP distance distribution was wide, indicating substantial diversity, two distinct low-SNP clusters were identified. One cluster comprised isolates that were genomically indistinguishable at the core genome level, while the second comprised isolates differing by only 0–3 SNPs. These patterns are consistent with recent transmission or shared-source acquisition within the ward. ^46^ Importantly, however, these clusters were observed against a backdrop of many genetically unrelated isolates, indicating that ward-level transmission is only part of the overall picture. This mixed scenario suggests that ESBL-producing *E. coli* in the orthopaedic ward likely arise through both ongoing circulation of closely related strains and repeated introduction of unrelated lineages from colonized patients entering the hospital. Such a pattern is epidemiologically plausible in a setting where patient turnover, referral pathways, and prior healthcare exposure may continually seed new resistant strains into the ward environment ( Figure 1).

**Figure 1.**
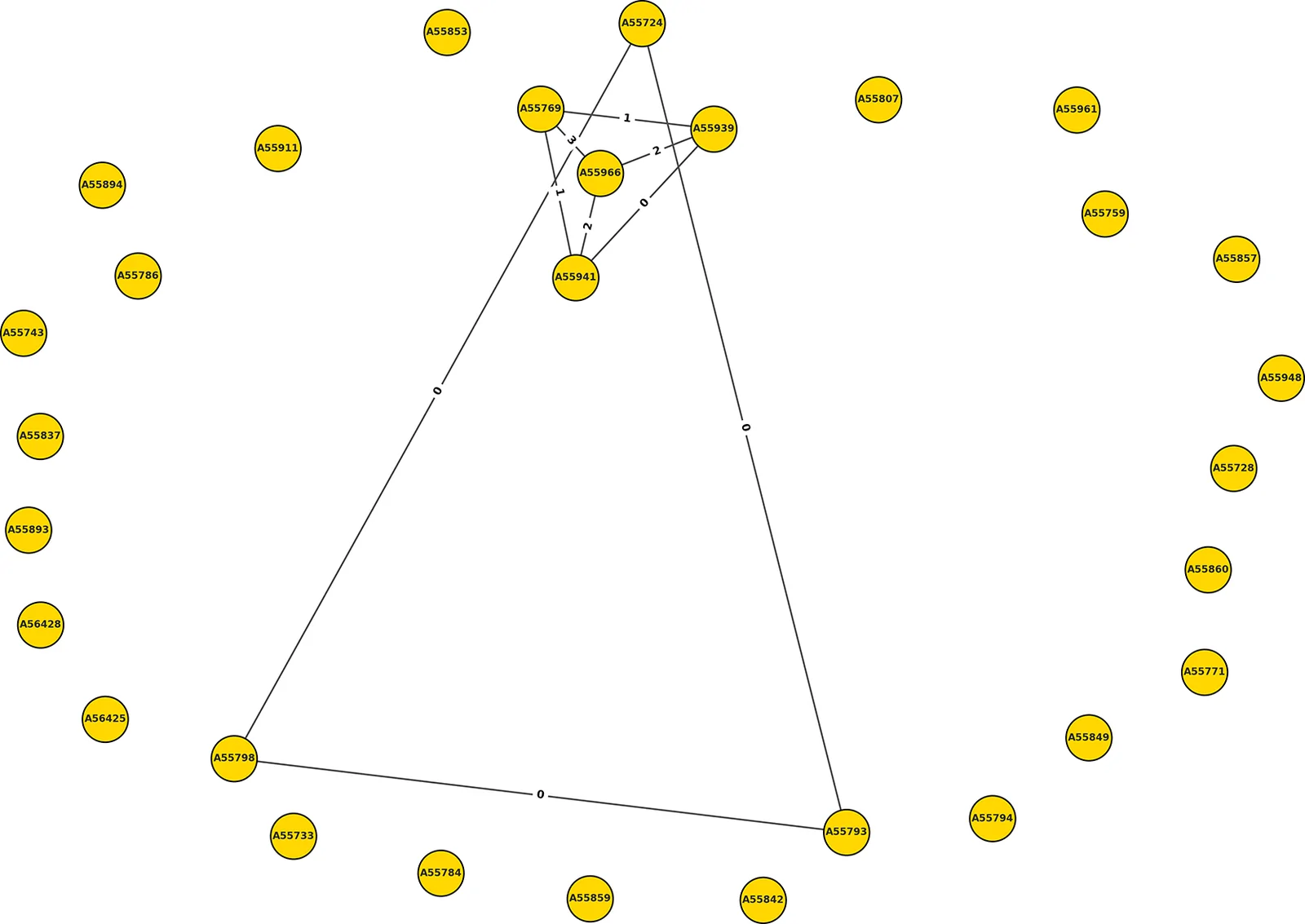
Transmission-linked network of ESBL-producing *E. coli* isolates (≤5 SNPs). Nodes represent individual isolates, and edges represent pairwise SNP distances of ≤5 SNPs derived from recombination-filtered core genome analysis. Edge labels indicate the number of SNP differences between connected isolates. Two transmission clusters were identified. The first cluster comprised isolates A55939, A55769, A55966, and A55941, with pairwise SNP distances ranging from 0 to 3 SNPs, consistent with recent transmission or a shared source. The second cluster comprised isolates A55724, A55793, and A55798, which were genetically indistinguishable at the core genome level (0 SNP differences). Most isolates were not connected within the ≤5 SNP threshold, indicating substantial genetic diversity and suggesting multiple independent introductions into the orthopaedic ward.

**Figure 2.**
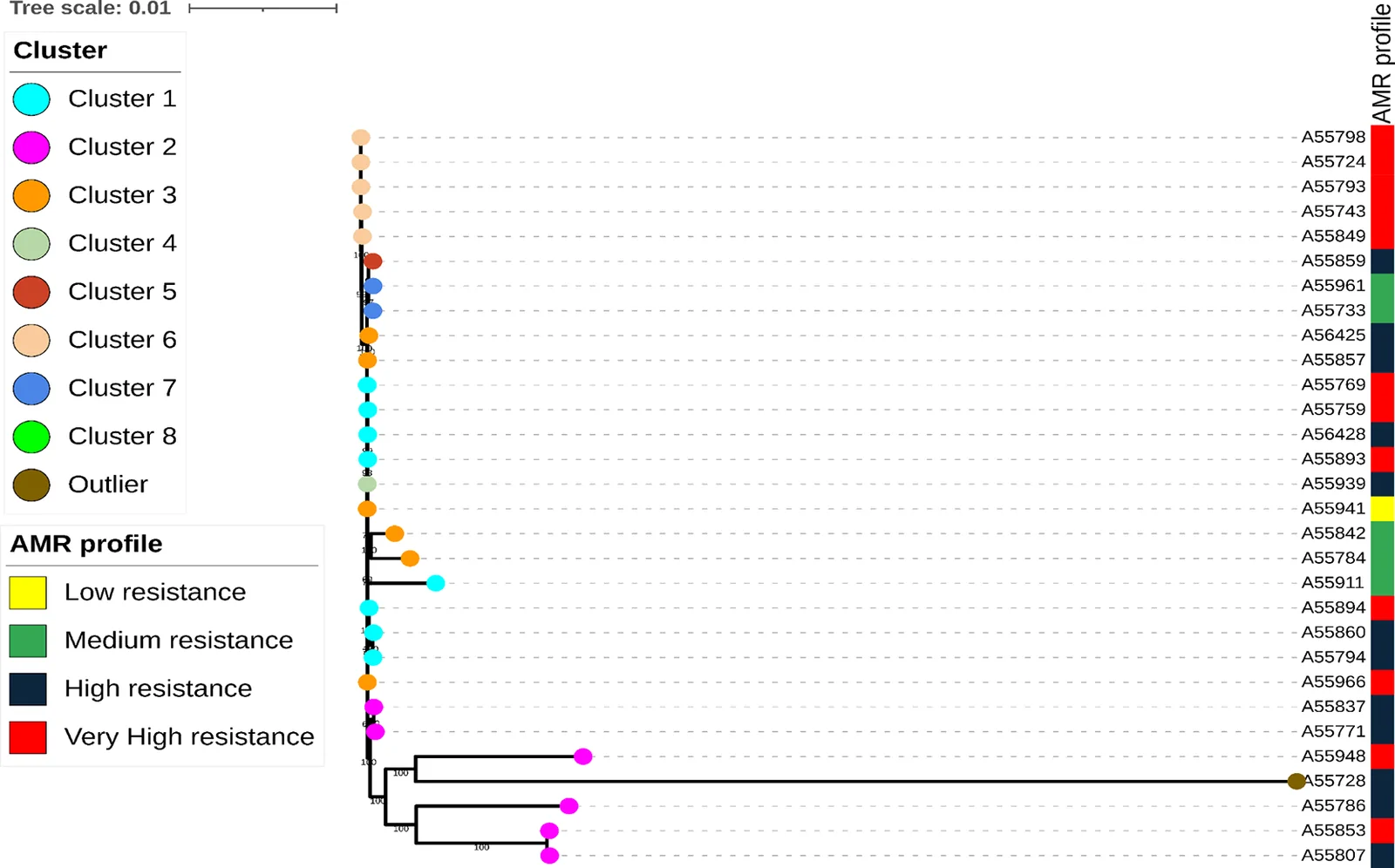
Core genome phylogenetic relationships and antimicrobial resistance profiles of ESBL-producing *E. coli* isolates. Core genome SNP-based phylogenetic tree of 39 ESBL-producing *E. coli* isolates recovered from stool or rectal swabs of patients admitted to an orthopaedic ward in Mwanza, Tanzania. The tree was constructed from a recombination-filtered core genome alignment generated using Snippy, with recombination regions identified and masked using Gubbins, and maximum-likelihood inference performed using IQ-TREE. Isolates are grouped into eight phylogenetic clusters (Clusters 1–8) and one genetically distinct outlier, indicated by colored symbols at the tips. Several clusters comprise closely related isolates with short branch lengths, consistent with low pairwise SNP distances and potential recent transmission events, while others show greater divergence, reflecting multiple independent lineages circulating within the ward.

The recombination-filtered phylogeny and shared variant analysis support this interpretation. Several isolates formed tight clusters with short branches and shared variant profiles, consistent with recent common ancestry and possible recent transmission. In contrast, isolates such as A55728 showed marked divergence from the main clusters, reflecting distant ancestry and arguing against participation in recent local transmission events. These observations highlight the value of combining phylogenetic structure, SNP distances, and shared variant patterns when interpreting transmission dynamics. In a setting such as this, reliance on one metric alone could oversimplify the epidemiology; instead, the data support a model in which both clonal spread and genomic convergence through shared resistance elements are occurring simultaneously. ^16, 18, 26^

From a clinical and public health perspective, these findings have several implications. These findings highlight the importance of colonized patients as reservoirs of multidrug-resistant organisms with potential implications for infection prevention and antimicrobial stewardship. First, they reinforce the importance of gastrointestinal colonization surveillance in hospitalized patients, particularly in high-risk wards such as orthopaedics, where colonized patients may serve as reservoirs for onward transmission and possibly subsequent infection. ^11, 14^ Second, they support the need for strengthened infection prevention and control measures aimed not only at preventing environmental contamination and patient-to-patient spread, but also at recognizing the role of patient importation of resistant strains. ^25^ Third, they highlight the utility of genomic surveillance for distinguishing between true clonal spread and broader background diversity, thereby informing targeted interventions. In resource-constrained settings, this kind of genomic information may be especially valuable for prioritizing IPC strategies and antimicrobial stewardship around the organisms and lineages most likely to circulate and persist. ^26^

This study has limitations that should be acknowledged. First, only 39 isolates passed sequencing quality control and were included in the genomic analyses, which limits the breadth of inference. Second, the study focused on patient-derived stool and rectal swab isolates and did not include contemporaneous sequencing of environmental or healthcare worker isolates, limiting our ability to reconstruct precise transmission pathways. Third, the analysis was conducted within a single orthopaedic ward at one tertiary hospital, and the findings may therefore not be fully generalizable to other wards, hospitals, or regions. Fourth, although plasmid replicons and resistance genes were identified, the use of short-read sequencing limits full resolution of plasmid architecture and the exact genomic context of some resistance determinants. Finally, while low SNP thresholds provide evidence consistent with recent transmission, genomic similarity alone cannot establish the direction or exact route of transmission in the absence of denser epidemiological linkage data. These limitations mean that our findings should be interpreted as evidence of likely circulation patterns rather than definitive proof of specific transmission events.

Despite these limitations, this study provides important genomic evidence that ESBL-producing *E. coli* colonizing orthopaedic patients in Mwanza comprise a multidrug-resistant, genomically diverse, yet epidemiologically connected population. The co-circulation of high-risk international clones, widespread *bla*_CTX-M-15_, common IncF-family plasmids, and low-SNP transmission clusters suggests that the orthopaedic ward functions as both a point of convergence for imported resistant strains and a setting that may permit local persistence and spread. These findings underscore the need to integrate genomic surveillance into infection prevention and antimicrobial resistance monitoring frameworks in sub-Saharan African hospitals, particularly in wards where prolonged stays and antibiotic exposure may amplify the risks of colonization and onward transmission. ^6, 26^

## Conclusions

This study demonstrates that ESBL-producing *E. coli* colonizing patients in an orthopaedic ward in Mwanza, Tanzania comprise a multidrug-resistant, genomically diverse population dominated by *bla*_CTX-M-15_ and carried across multiple globally recognized and locally circulating lineages. Despite this overall diversity, the identification of low-SNP transmission clusters indicates that recent transmission or shared-source exposure is occurring within the ward, highlighting the coexistence of multiple introductions and localized spread.

The widespread presence of IncF-family plasmids and shared resistance determinants across phylogenetically distinct isolates suggests that horizontal gene transfer, alongside clonal expansion, plays a key role in shaping the resistance landscape. The concurrent detection of virulence-associated genes further underscores the clinical significance of these colonizing strains as potential sources of difficult-to-treat infections.

Together, these findings emphasize the importance of gastrointestinal colonization as a reservoir for antimicrobial resistance in hospitalized patients and highlight the need for strengthened infection prevention and control measures, improved antimicrobial stewardship, and the integration of genomic surveillance into routine monitoring frameworks in resource-limited healthcare settings.

## Software availability

The bioinformatics analyses in this study were conducted using the rMAP-2.0 (Rapid Microbial Analysis Pipeline). The pipeline is openly available and can be accessed via GitHub:

• Repository: https://github.com/gmboowa/rMAP-2.0
• Version: v2.0
• License: MIT License

A representative report including sample quality control (QC) metrics and downstream analysis outputs is available at: https://gmboowa.github.io/rMAP-2.0/reports/esbl_ecoli.html

## Ethics approval and consent to participate

This study was conducted in accordance with the Declaration of Helsinki and relevant national regulations. It was approved by the Joint CUHAS/BMC Research and Ethics Committee (CREC/409/2019) and the National Health Research Ethics Review Committee of the National Institute for Medical Research (NIMR/HQ/R.8a/Vol. IX/3322) in Tanzania. Permission to conduct the study was obtained from the hospital administration, the Head of the Department of Orthopaedics, and ward supervisors/in-charge nurses prior to sample and data collection.

Sampling did not involve collection of identifiable patient information. In the parent study, written informed consent (or assent for children) was obtained from all participants where clinical samples and data were collected. Patients with suspected surgical site infections received appropriate clinical management, including culture and antimicrobial susceptibility testing to guide therapy.

## Data Availability

All sequencing data generated in this study have been deposited in the National Center for Biotechnology Information under BioProject accession PRJNA1452460. Raw sequencing reads for all *E. coli* isolates are available in the Sequence Read Archive and are linked to this BioProject. The corresponding BioSample accession numbers are SAMN57232750–SAMN57232788, and these are also listed in the Extended data 1 (Table S1) associated with this study. Bioinformatics analyses were performed using reproducible workflows implemented in rMAP-2.0 (Rapid Microbial Analysis Pipeline). The scripts and workflow used for data processing and analysis are available at https://github.com/gmboowa/rMAP-2.0. The following Extended dataset is provided:
•
**Extended data 1 (Table S1)**: Metadata for all isolates, including BioSample accessions, sample information, and associated genomic characteristics. This dataset is available on Figshare: https://doi.org/10.6084/m9.figshare.32016402 Internal isolate identifiers used in the manuscript are not included in the public dataset to ensure de-identification; corresponding SRA accession numbers are provided in Extended data Table S1. All data are openly available without restriction in accordance with F1000Research open data policies.
