# Genomic characterization of ESBL-producing *Escherichia coli* isolates from patients in an orthopaedic ward in Mwanza, Tanzania

**DOI:** 10.12688/f1000research.180294.2

**Published:** 2026-06-25

**Authors:** Gerald Mboowa, Benson R Kidenya, Ivan Sserwadda, Stephen Kanyerezi, Jeremiah Seni

**Affiliations:** 1 Department of Immunology and Molecular Biology, College of Health Sciences, School of Biomedical Sciences, Makerere University, Kampala, P.O. Box 7072, Uganda; 2 Weill Bugando School of Medicine, Catholic University of Health and Allied Sciences, Mwanza, P.O. Box 1464, Tanzania

**Keywords:** Escherichia coli, ESBL (extended-spectrum β-lactamase), antimicrobial resistance, hospital environment, whole-genome sequencing

## Abstract

**Background:**

Extended-spectrum β-lactamase (ESBL)-producing *Escherichia coli* are a major contributor to antimicrobial resistance (AMR) in healthcare settings, yet their genomic diversity and population structure remain poorly characterized in low-resource settings. Orthopaedic wards may facilitate persistence, acquisition, and possible onward spread of resistant organisms due to prolonged hospitalization, frequent antimicrobial exposure, and increased patient vulnerability. This study investigated the genomic characteristics of ESBL-producing *E. coli* recovered from hospitalized patients in an orthopaedic ward in Mwanza, Tanzania.

**Methods:**

We performed whole-genome sequencing and comparative genomic analysis of ESBL-confirmed *E. coli* isolates obtained from stool or rectal swabs of hospitalized patients. Sequence reads were quality controlled and assembled using standard pipelines. Core genome single nucleotide polymorphism (SNP) alignments were generated using Snippy, and recombination was identified and masked using Gubbins prior to phylogenetic reconstruction. Sequence types (STs), phylogroups, predicted serotypes, putative pathotype-associated marker profiles, AMR determinants, plasmid replicons, and virulence-associated genes were identified using established bioinformatics tools and databases.

**Results:**

Phylogenetic analysis revealed a polyclonal population structure comprising multiple distinct lineages rather than a single outbreak clone. Several isolates formed low-SNP clusters compatible with recent shared ancestry, possible shared-source exposure, or localized circulation within the ward. The population included internationally recognized sequence types, including ST131, ST1193, ST69, ST617, and ST38. Phylogroup analysis showed a diverse distribution, with phylogroup A most common, followed by B1, B2, D, U/unassigned, C, F, and one cryptic lineage. ESBL production was predominantly associated with *bla*_CTX-M-15_, detected in most isolates, alongside additional resistance determinants to aminoglycosides, quinolones, sulfonamides, trimethoprim, and tetracyclines. IncF-family plasmid replicons predominated, with additional IncY plasmids identified. Virulence-associated genes, including *fdeC* and *ybtP/ybtQ*, were widely distributed.

**Conclusions:**

ESBL-producing *E. coli* in this orthopaedic ward represent a genomically diverse, multidrug-resistant population dominated by *bla*_CTX-M-15_-positive lineages. The coexistence of multiple lineages alongside low-SNP clusters suggests repeated introductions with possible localized circulation; however, genomic similarity alone cannot establish direct transmission. These findings highlight colonized patients as reservoirs of AMR and underscore the need to integrate genomic surveillance into infection prevention and control strategies in resource-constrained healthcare settings.

## Introduction

Antimicrobial resistance (AMR) among Gram-negative bacteria represents a major global public health threat, with extended-spectrum *β*-lactamase (ESBL)-producing *Escherichia coli* emerging as one of the most clinically significant pathogens in both community and healthcare settings. ^1, 2^ ESBL enzymes, particularly those of the CTX-M family, confer resistance to third-generation cephalosporins and are frequently co-associated with resistance to other antimicrobial classes, resulting in multidrug-resistant (MDR) phenotypes that complicate treatment and increase morbidity and mortality. ^3– 5^ Among these, *bla*_CTX-M-15_ has become the dominant ESBL gene globally and is widely disseminated across diverse *E. coli* lineages and geographic regions. ^6, 7^

In sub-Saharan Africa, the burden of ESBL-producing Enterobacteriaceae is increasing, driven by limited diagnostic capacity, high antimicrobial usage, and constrained infection prevention and control (IPC) infrastructure. ^8– 10^ *E. coli* is a major contributor to this burden, not only as a cause of invasive infections but also as a colonizer of the gastrointestinal tract, where it serves as a reservoir for AMR genes. ^11, 12^ Colonization is particularly important in hospitalized patients, as it can precede infection and facilitate onward transmission within healthcare settings. ^12– 20^ However, genomic data describing the population structure, resistance determinants, and transmission dynamics of ESBL-producing *E. coli* in African hospital environments remain limited.

Orthopaedic wards represent a high-risk setting for the persistence and dissemination of MDR organisms. ^21– 24^ Patients in these settings often experience prolonged hospital stays, repeated exposure to antibiotics, and increased vulnerability due to underlying trauma or surgical interventions. ^25^ These factors create conditions that may facilitate the maintenance and spread of resistant bacterial populations within the patient cohort. Understanding the diversity and relatedness of colonizing strains in such settings is therefore essential for informing infection prevention and antimicrobial stewardship strategies.

Whole-genome sequencing (WGS) has transformed the study of bacterial epidemiology by enabling high-resolution characterization of population structure, resistance mechanisms, and clonal relationships. ^26^ Genomic approaches allow discrimination between clonal expansion and polyclonal populations, identification of high-risk sequence types such as ST131 and ST1193, and detection of mobile genetic elements, including plasmids that mediate the spread of AMR genes. ^27– 29^ Despite these advances, relatively few studies have applied WGS to investigate ESBL-producing *E. coli* colonization in hospitalized patient populations in sub-Saharan Africa.

Data from Tanzanian healthcare settings are particularly scarce, especially with respect to genomic characterization of ESBL-producing *E. coli* in high-risk hospital wards. In this study, we performed a genomic epidemiological analysis of ESBL-confirmed *E. coli* isolates recovered from stool or rectal swabs of patients admitted to an orthopaedic ward in a tertiary hospital in Mwanza, Tanzania. By integrating WGS with available non-identifiable clinical and ward-level metadata, we aimed to (i) characterize the population structure and sequence types of ESBL-producing *E. coli*, (ii) define their AMR, plasmid, virulence-associated profiles, phylogroup, serotype, and putative pathotype-associated marker profiles and (iii) assess patterns of genomic relatedness consistent with the circulation of multidrug-resistant lineages within the patient population.

## Methods

### Study setting and design

This study was nested within a larger longitudinal investigation conducted over a 4-month period, from 3 January 2020 to 30 May 2020, assessing gastrointestinal colonization with ESBL-producing Enterobacteriaceae among orthopaedic patients admitted to a tertiary hospital in Mwanza, Tanzania. ^30^ Although the parent study included broader ward-level sampling activities, the present whole-genome sequencing analysis was restricted to patient-derived stool or rectal swab isolates. For this study, we analyzed ESBL-confirmed *E. coli* isolates recovered from stool or rectal swabs collected from patients admitted to the orthopaedic ward. By integrating whole-genome sequencing data with available non-identifiable clinical and ward-level metadata, the study aimed to investigate the population structure of ESBL-producing *E. coli*, define their AMR and associated genomic features, and assess patterns of genomic relatedness consistent with recent shared ancestry, possible shared-source exposure, or localized ward-level circulation of multidrug-resistant lineages.

### Bacterial isolation and ESBL confirmation

Patient-derived stool and rectal swab samples were processed using standard microbiological procedures at the Department of Microbiology and Immunology, Weill Bugando School of Medicine, Catholic University of Health and Allied Sciences, Mwanza, Tanzania. Samples were inoculated onto MacConkey agar (Oxoid, UK) and CHROMagar™ ESBL (CHROMagar, France), a selective medium containing cephalosporins for the isolation of ESBL-producing Gram-negative bacteria. Plates were incubated aerobically at 35–37 °C for 18–24 hours. Colonies with morphology consistent with *E. coli* were selected and subcultured to obtain pure isolates. ESBL production was then confirmed phenotypically using the combination disk method, comparing inhibition zones for cefotaxime and ceftazidime, alone and in combination with clavulanic acid, in accordance with CLSI recommendations for phenotypic ESBL confirmation. ^31^ Only patient-derived isolates confirmed phenotypically as ESBL-producing *E. coli* were included in downstream genomic analyses.

### DNA extraction, library preparation, and sequencing

Pure patient-derived *E. coli* isolates were shipped to the Genomics Laboratory, Department of Immunology and Molecular Biology, College of Health Sciences, Makerere University, Kampala, Uganda, where genomic DNA was extracted using standardized protocols. DNA concentration was measured using the Qubit dsDNA High Sensitivity Assay (Thermo Fisher Scientific, USA), and DNA purity was assessed using a NanoDrop spectrophotometer (Thermo Fisher Scientific, USA) by evaluating A260/280 and A260/230 ratios. DNA integrity was further assessed by 1.0% agarose gel electrophoresis. Whole-genome sequencing was performed at the Earlham Institute (Norwich, UK) using the Illumina NovaSeq 6000 platform. Sequencing libraries were prepared using the LITE (Low Input Transposase-Enabled) protocol, and sequencing was carried out using paired-end 150-bp chemistry (2 × 150 bp reads).

### Read processing and genome assembly

Raw sequencing reads were subjected to quality control using FastQC (v0.12.1), ^32^ followed by adapter trimming and removal of low-quality bases using Trimmomatic (v0.40), ^33^ with default parameters. High-quality reads were retained for downstream analyses. *De novo* genome assemblies were generated using SKESA (Strategic *K*-mer Extension for Scrupulous Assemblies) assembler (v2.4.0), ^34^ with default settings and assembly quality was assessed using standard metrics, including genome size, contiguity, and N50 values, to ensure suitability for comparative genomic analysis. Of the 45 ESBL-producing *E. coli* isolates initially selected for whole-genome sequencing, 39 met sequencing, assembly, and species-confirmation quality-control criteria and were retained for downstream comparative genomic analyses. Six isolates were excluded because they did not meet one or more QC criteria, including insufficient read yield, excessive assembly fragmentation, atypical assembly metrics, or incomplete species-confirmation evidence. Isolate-level sequencing and assembly metrics, including read counts, estimated coverage depth, number of contigs, N50, genome size, GC content, and QC status, are provided in Extended Data Table S1.

### Genomic species confirmation

Genomic species confirmation was performed using a multi-tool approach to support accurate taxonomic classification and to ensure that only confirmed *E. coli* genomes were retained for downstream analyses. Draft genome assemblies were analyzed using GTDB-Tk (v2.6.0) ^35^ (Genome Taxonomy Database Toolkit), which assigns taxonomy based on genome-wide phylogenetic placement against the GTDB reference database.

To complement this, GAMBIT v1.1.0 (Genomic Approximation Method for Bacterial Identification and Tracking) ^36^ was used to provide *k*-mer–based taxonomic classification, enabling rapid and high-resolution identification of bacterial genomes. In addition, Basic Local Alignment Search Tool for nucleotides (BLASTn) searches were performed against the NCBI nucleotide database to confirm species identity based on sequence similarity to well-characterized reference genomes. Blast analyses were conducted using the Docker image gmboowa/blast-analysis:1.9.4.

Concordance across these three independent approaches (GTDB-Tk, GAMBIT, and BLAST) was used to validate species assignments. Isolates with incomplete or inconsistent species-confirmation evidence were not retained for downstream comparative genomic analyses.

### Genome characterization and typing

Sequence types (STs) were assigned from draft genome assemblies using the MLST module in rMAP-2.0, implemented with the mlst tool (Torsten Seemann; Docker image staphb/mlst:2.19.0). This approach scans assembled contigs against PubMLST-curated schemes to determine the sequence type and allele profile for each isolate, enabling classification into globally recognized *E. coli* lineages. ^37^ In addition to multilocus sequence typing, E. coli phylogroups were assigned *in silico* using EzClermont. Predicted O serotypes were determined using ECTyper and SerotypeFinder, while putative pathotype-associated marker profiles and virulence-associated genes were summarized from ECTyper, VirulenceFinder, and AMRFinderPlus outputs. Because pathotype assignment from genome data alone can be uncertain, particularly when virulence-marker profiles are incomplete or overlapping, these results were interpreted as putative genomic pathotype-associated marker profiles rather than definitive phenotypic pathotype classifications. Isolate-level sequence type, phylogroup, predicted serotype, putative pathotype-associated marker profile, virulence-marker results, and sequencing/assembly quality metrics are provided in Extended Data Table S1.

### Read mapping and variant calling

Quality-controlled reads were mapped to the clinical *E. coli* reference genome GCF_000285655.3_EC958.v1 using the Snippy pipeline (v4.6.0), ^38^ which integrates BWA-MEM for read alignment and bcftools for variant calling. This clinically relevant reference was selected to provide a consistent coordinate framework for read mapping and SNP calling across the isolate collection. Core genome single nucleotide polymorphisms (SNPs) were identified across all isolates, and a multiple sequence alignment of core SNPs was generated for downstream phylogenetic analysis.

### Recombination filtering and phylogenetic reconstruction

To account for homologous recombination, SNP alignments were processed using Gubbins v3.4.1 (Genealogies Unbiased By recomBinations In Nucleotide Sequences), ^39^ which identifies and masks regions of elevated SNP density consistent with recombination. The resulting recombination-filtered alignment represents clonal variation across the dataset. Maximum-likelihood phylogenetic trees were inferred using IQ-TREE v3.1.1, ^40^ based on the recombination-filtered core genome SNP alignment. Branch support was assessed using standard bootstrap approaches. Pairwise SNP distances between isolates were calculated using snp-dists, providing a quantitative measure of genomic relatedness. The final phylogenetic tree was midpoint-rooted for visualization and displayed in iTOL v7.0 (Interactive Tree of Life), ^41^ where isolate metadata, including sequence type, phylogroup, and AMR profile, were incorporated to annotate the tree and facilitate interpretation of clustering patterns, genomic relatedness, and possible ward-level circulation.

### Transmission network analysis

To investigate potential transmission events among ESBL-producing *E. coli* isolates, pairwise SNP distances were calculated from the recombination-filtered core genome alignment generated using Snippy and processed with Gubbins. SNP distances were computed using snp-dists, providing a matrix of pairwise genomic distances among the 39 clinical isolates and the included reference genome. A low-SNP relatedness network was constructed using a predefined SNP threshold of ≤5 SNPs, consistent with thresholds commonly applied to infer recent transmission or shared-source acquisition in bacterial genomic epidemiology. Isolates and the reference genome were represented as nodes, and edges were drawn between nodes with pairwise SNP distances at or below this threshold. Edge labels indicate the number of SNP differences between connected nodes. The network was generated using Python (version 3.14) with the NetworkX library for graph construction and Matplotlib for visualization. A force-directed layout algorithm (spring layout) was applied to position nodes based on their connectivity, enabling visualization of low-SNP relatedness clusters among closely related isolates.

### Antimicrobial resistance, plasmid, and virulence analysis

Antimicrobial resistance genes were identified using the ResFinder v4.5.0 database, ^42^ enabling classification of resistance determinants into antimicrobial classes. MDR was defined as the presence of resistance determinants spanning three or more antimicrobial classes. ^43^ Plasmid replicon markers were identified within rMAP-2.0 using PlasmidFinder ^44^ to screen assembled genomes for known plasmid replicon sequences, while virulence-associated genes were detected using ABRicate, ^45^ implemented through the Docker image staphb/abricate:1.0.0 against curated *E. coli* virulence factor databasesPlasmid. ^37^

### Data integration and comparative analysis

Genomic data were integrated with available non-identifiable clinical and ward-level metadata, including sample source and collection timing where available. This enabled integrated analysis of population structure, antimicrobial resistance burden, plasmid replicon content, virulence-associated profiles, and phylogenetic clustering. Because temporal, spatial, environmental, and patient-contact metadata were limited, phylogenetic clustering was interpreted cautiously as evidence consistent with genomic relatedness, recent shared ancestry, possible shared-source exposure, or localized ward-level circulation, rather than as definitive evidence of direct transmissionGenomic.

## Results

### Sample characteristics

A total of 45 ESBL-producing clinical *E. coli* isolates were initially collected from patient-derived stool and rectal swab samples obtained from individuals admitted to the orthopaedic ward. Of these, 39 isolates passed quality control and were included in the whole-genome sequencing and downstream analyses ( Table 1). Core genome phylogenetic analysis revealed multiple distinct clusters, indicating genomic diversity among isolates circulating within the ward. Several isolates formed tight phylogenetic clusters, suggestive of potential shared sources, while others appeared more genetically distinct. Notably, isolate A55728 formed a long independent branch, indicating substantial divergence from other isolates. The distribution of isolates across phylogenetic clusters, together with metadata on sample source and collection site, provided a framework for investigating potential transmission dynamics. Detailed isolate-level characteristics are presented in **Extended data 1 (Table S1)**.

**Table 1.** Summary of patient-derived ESBL-producing *E. coli* isolates included in whole-genome sequencing analysis.

Characteristic	Value
Total isolates	39
Source	Patient-derived stool/rectal swab isolates only
Median age (years)	30.5 years
Sex distribution	21 female (53.8%), 18 male (46.2%)
Common fracture types	Closed (66.7%), Open (23.1%)
Most common fracture sites	Femur (38.5%), Tibia (10.3%)
Median genome size (Mb)	~5.1
Median contigs	90 (range: 50–3339)
Median GC (%)	~50.6
MDR isolates	39/39 (100%)
Dominant sequence types (MLST)	ST131, ST1193, ST69
Number of phylogenetic clusters	8 clusters + 1 outlier

### Genomic features

Whole-genome sequencing of 39 ESBL-producing *E. coli* isolates generated high-quality draft assemblies suitable for downstream comparative genomic analysis. Species identity was genomically confirmed using a combination of GTDB-Tk, GAMBIT, and BLAST-based approaches, supporting accurate taxonomic assignment within the *E. coli* species complex. Isolate-level taxonomic confirmation and assembly metrics are provided in Extended Data Table S1.

The assemblies had a median genome size of approximately 5.1 Mb, consistent with typical *E. coli* genomes. Assembly fragmentation varied across isolates, with a median of 90 contigs (range: 50–3339), indicating overall good assembly quality, although a small number of genomes were highly fragmented. The GC content was highly conserved across isolates, with a median of approximately 50.6%, reflecting the expected genomic composition of *E. coli.*

Sequence typing revealed a genetically diverse population comprising several globally recognized high-risk and community-associated lineages, including ST131, ST1193, ST69, ST617, and ST38, alongside additional less frequent sequence types. This diversity indicates the co-circulation of internationally disseminated high-risk clones and locally established lineages within the orthopaedic ward population.

Core genome SNP analysis using Snippy identified substantial genomic diversity across the isolates. The reference-based alignment revealed a wide range of variant sites, highlighting the presence of both closely related and highly divergent isolates within the same clinical setting. The distribution of SNPs per isolate demonstrated marked heterogeneity, with some isolates showing minimal divergence compatible with recent shared ancestry, possible shared-source exposure, or localized ward-level circulation, while others exhibited extensive polymorphism, reflecting the presence of multiple independent lineages.

### Antimicrobial resistance, plasmids, and virulence determinants

All 39 isolates were classified as MDR, carrying resistance determinants to three or more antimicrobial classes. ESBL production was predominantly associated with *bla*_CTX-M-15_, which was detected in most isolates and is consistent with its recognized role as a globally disseminated ESBL determinant.

Beyond *β*-lactam resistance, the isolates harbored genes associated with resistance to several additional antimicrobial classes, including aminoglycosides, fluoroquinolones, sulfonamides, trimethoprim, and tetracyclines. This broad resistome highlights the accumulation of multiple resistance determinants within individual isolates and reflects the complex AMR burden present in the orthopaedic ward setting.

Plasmid replicon analysis showed a predominance of IncF-family replicon markers, which are frequently implicated in the dissemination of ESBL genes in *E. coli.*. Additional plasmid replicon markers, including IncY, were also identified in a subset of isolates, indicating the possible involvement of diverse mobile genetic elements in the acquisition and dissemination of resistance determinants.

Virulence-associated genes were also widely distributed across the isolate collection, including factors linked to adhesion, iron acquisition, and extraintestinal survival, such as *fdeC* and *ybtP/ybtQ.* The co-occurrence of antimicrobial resistance and virulence-associated determinants underscores the clinical relevance of these isolates and raises concern about the circulation of lineages with both enhanced pathogenic potential and limited treatment options. Isolate-level AMR determinants, plasmid replicon markers, virulence-associated genes, and available genotype—phenotype interpretation are provided in Extended Data Table S1.

### Pairwise SNP distances and transmission inference

The pairwise SNP analysis adds an important epidemiological dimension to these genomic findings. Although the overall SNP distance distribution was wide, indicating substantial diversity, two distinct low-SNP clusters were identified. One cluster comprised isolates that were genomically indistinguishable at the core genome level, while the second comprised isolates differing by only 0–3 SNPs. These patterns are compatible with recent shared ancestry, possible recent transmission, or shared-source acquisition within the ward. ^46^ Importantly, however, these clusters were observed against a backdrop of many genetically unrelated isolates, indicating that low-SNP relatedness is only part of the overall genomic picture. This mixed scenario suggests that ESBL-producing *E. coli* in the orthopaedic ward likely arise through both ongoing circulation of closely related strains and repeated introduction of unrelated lineages from colonized patients entering the hospital. Such a pattern is epidemiologically plausible in a setting where patient turnover, referral pathways, and prior healthcare exposure may introduce new resistant strains into the ward population ( Figure 1).

**Figure 1.**
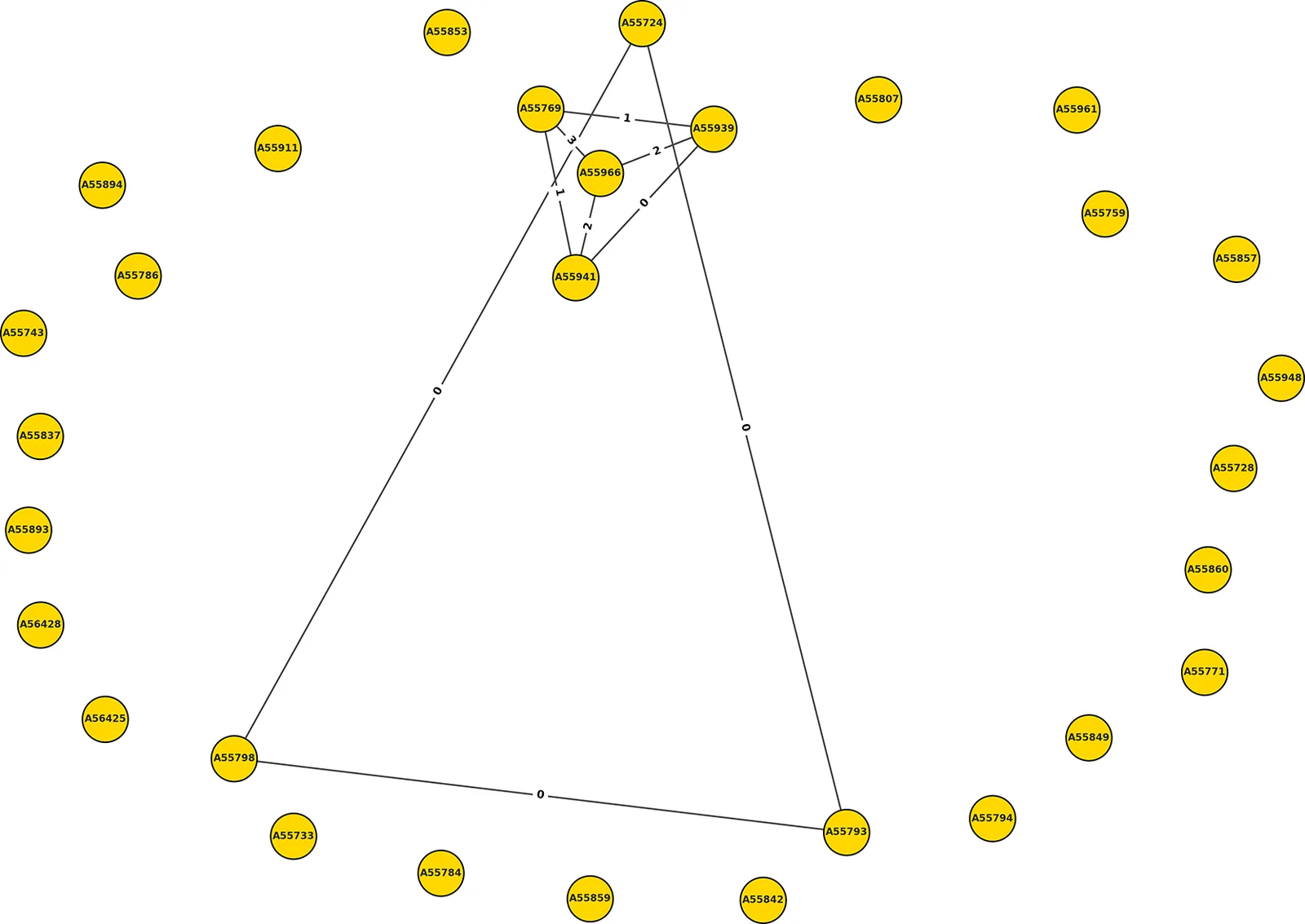
Low-SNP relatedness network of ESBL-producing *E. coli* isolates (≤5 SNPs). Nodes represent individual isolates, and edges represent pairwise SNP distances of ≤5 SNPs derived from the recombination-filtered core genome alignment. Edge labels indicate the number of SNP differences between connected isolates. Two low-SNP clusters were identified. The first cluster comprised isolates A55939, A55769, A55966, and A55941, with pairwise SNP distances ranging from 0 to 3 SNPs. The second cluster comprised isolates A55724, A55793, and A55798, which were genetically indistinguishable at the core genome level with 0 SNP differences. These low-SNP clusters are compatible with recent shared ancestry, possible recent transmission, or shared-source exposure, but genomic similarity alone cannot confirm direct transmission, identify the transmission route, or infer directionality without supporting temporal, spatial, patient-contact, and environmental sampling data. Most isolates were not connected within the ≤5 SNP threshold, indicating substantial genetic diversity and suggesting multiple independent introductions into the orthopaedic ward.

### Identification of genetically indistinguishable isolates (0 SNP cluster)

A cluster of isolates with zero SNP differences was identified, comprising isolates A55724, A55793, and A55798. These isolates were genetically indistinguishable at the core genome level ( Table 2), supporting recent shared ancestry, possible recent transmission, or a common-source exposure. However, in the absence of dense temporal, spatial, patient-contact, and environmental sampling data, direct or near-direct transmission cannot be confirmed from genomic similarity alone.

**Table 2.** Zero-SNP isolate pairs. Pairwise SNP distances were calculated from the recombination-filtered core genome alignment. These isolate pairs were genetically indistinguishable at the core genome level and are compatible with recent shared ancestry, possible recent transmission, or shared-source exposure. Genomic similarity alone cannot confirm direct transmission or identify the transmission route without supporting epidemiological data.

Isolate 1	Isolate 2	SNP Distance
A55724	A55793	0
A55724	A55798	0
A55793	A55798	0

### Detection of a closely related low-SNP cluster (≤3 SNPs)

A second cluster of closely related isolates was identified, with pairwise SNP distances ranging from 0 to 3 SNPs. This cluster included isolates A55769, A55939, A55941, and A55966 ( Table 3). The low level of genomic variation within this group is compatible with recent shared ancestry, possible recent transmission, or shared-source exposure within the ward; however, the direction and route of transmission cannot be established without additional temporal, spatial, patient-contact, and environmental sampling data.

**Table 3.** Closely related low-SNP isolate pairs. Pairwise SNP distances were calculated from the recombination-filtered core genome alignment. These isolate pairs differed by 0–3 SNPs and are compatible with recent shared ancestry, possible recent transmission, or shared-source exposure. Genomic similarity alone cannot confirm direct transmission or identify the transmission route without supporting epidemiological data.

Isolate 1	Isolate 2	SNP distance
A55769	A55939	1
A55769	A55941	1
A55769	A55966	3
A55939	A55941	0
A55939	A55966	2
A55941	A55966	2

### Interpretation of transmission dynamics

Together, these findings indicate a mixed epidemiological scenario within the orthopaedic ward. The presence of genetically indistinguishable isolates and closely related low-SNP clusters supports recent shared ancestry, possible recent transmission, or shared-source exposure, while the broader distribution of high SNP distances across the dataset reflects a diverse background population structure likely driven by multiple introductions of unrelated *E. coli* lineages into the ward. These results demonstrate the coexistence of low-SNP clusters and genetically diverse strains, underscoring the importance of integrating genomic data with temporal, spatial, patient-level, and epidemiological context to better understand possible circulation patterns and inform infection prevention strategies.

Two distinct low-SNP clusters were identified based on pairwise SNP distances of ≤5 SNPs. The first cluster, comprising isolates A55724, A55793, and A55798, showed 0 SNP differences, indicating genetically indistinguishable isolates compatible with recent shared ancestry, possible recent transmission, or a common-source exposure. The second cluster, comprising A55769, A55939, A55941, and A55966, showed pairwise SNP distances ranging from 0 to 3 SNPs, consistent with a very closely related group. These clusters should be interpreted cautiously because genomic similarity alone cannot establish direct transmission, identify the transmission route, or infer directionality without supporting temporal, spatial, patient-contact, and environmental sampling data.

### Shared variant analysis

Analysis of shared SNPs identified groups of isolates with common variant profiles, further supporting the phylogenetic structure observed in the core genome SNP analysis. Isolates that were closely related in the phylogeny shared a higher proportion of SNPs, forming distinct genomic clusters compatible with recent shared ancestry, possible shared-source exposure, or localized ward-level circulation. In contrast, isolates with few or no shared variants were more genetically distant, supporting the presence of multiple unrelated lineages within the study population.

### Phylogenetic relationships

Core genome phylogenetic analysis based on the recombination-filtered SNP alignment demonstrated a clearly polyclonal population structure among the 39 ESBL-producing *E. coli* isolates ( Figure 2). The isolates were distributed across multiple distinct lineages, with eight phylogenetic clusters identified alongside one genetically distinct outlier, rather than forming a single outbreak clone. These clusters represent genomic lineages or related groups within the ward population and should not be interpreted as confirmed transmission chains. This overall structure indicates substantial genomic diversity among patient-derived isolates recovered from the orthopaedic ward.

Despite this broad diversity, several isolates formed tight phylogenetic clusters characterized by short branch lengths, consistent with the low pairwise SNP distances observed and compatible with recent shared ancestry, possible recent transmission, or shared-source exposure within the ward ( Figure 2).

In contrast, a subset of isolates displayed long branches, indicating marked genetic divergence and supporting the presence of unrelated strains likely introduced independently into the hospital setting. Notably, isolate A55728 formed a long independent branch and remained clearly separated from the main clusters, consistent with the high SNP distances observed and arguing against its involvement in the identified low-SNP clusters.

Taken together, these findings suggest that the ESBL-producing *E. coli* population in the orthopaedic ward is shaped by both the presence of successful lineages and the possible contribution of shared mobile genetic elements, resulting in isolates that are phylogenetically diverse but convergent in their resistance profiles.

### Comparative analysis

Comparative genomic analysis revealed both heterogeneity and shared features across the ESBL-producing *E. coli* population. Although the isolates were distributed across multiple sequence types and phylogenetic clusters, many shared a common repertoire of antimicrobial resistance determinants, most notably *bla*_CTX-M-15_, indicating the widespread distribution of key ESBL-associated resistance genes across genetically distinct lineages.

Comparison of plasmid replicon content further showed that diverse sequence types frequently carried IncF-family replicon markers, supporting a possible role for IncF-associated mobile genetic elements in the dissemination of resistance determinants. At the same time, variation in accessory resistance and virulence-associated genes across isolates highlighted the presence of lineage-specific genomic features superimposed on a shared multidrug-resistant background.

Taken together, these findings suggest that the ESBL-producing *E. coli* population in the orthopaedic ward is shaped by both the presence of successful lineages and the possible contribution of shared mobile genetic elements, resulting in isolates that are phylogenetically diverse but convergent in their resistance profiles.

## Discussion

This study provides a genomic snapshot of ESBL-producing *E. coli* colonizing patients admitted to an orthopaedic ward in Mwanza, Tanzania, and demonstrates that this patient population harbors a diverse but highly resistant set of lineages. Rather than identifying a single dominant outbreak clone, our data revealed a polyclonal population structure composed of multiple globally recognized and locally circulating sequence types, including ST131, ST1193, ST69, ST617, and ST38. At the same time, the identification of closely related isolate clusters with pairwise distances of 0–3 SNPs indicates that, within this broader diversity, recent shared ancestry, possible recent transmission, or shared-source exposure may have occurred within the ward. Taken together, these findings suggest that ESBL-producing *E. coli* in this setting may reflect a combination of repeated introduction of diverse strains and possible localized ward-level circulation of selected lineages. ^16, 18^

The predominance of *bla*_CTX-M-15_ across the isolate collection is notable and consistent with its recognized role as the most widely disseminated ESBL determinant in *E. coli.* ^5, 6^ In this study, *bla*_CTX-M-15_ was distributed across multiple phylogenetically distinct backgrounds, indicating that the burden of ESBL production in the ward is not restricted to a single lineage. This pattern supports the view that successful resistance genes can become embedded in diverse strain backgrounds, thereby amplifying their epidemiological and clinical impact. ^3^ The fact that all sequenced isolates were multidrug resistant, with resistance determinants spanning *β*-lactams, aminoglycosides, fluoroquinolones, sulfonamides, trimethoprim, and tetracyclines, further emphasizes the limited therapeutic options associated with these colonizing strains and reinforces the clinical importance of gastrointestinal carriage as a reservoir of difficult-to-treat organisms. ^11, 12^

The plasmid replicon findings provide a possible mechanistic explanation for convergence in resistance profiles across otherwise unrelated lineages. The predominance of IncF-family replicon markers, together with the detection of additional replicon markers such as IncY, suggests that mobile genetic elements may contribute to shaping the ward resistome. IncF plasmids are particularly important in *E. coli* because of their well-established association with ESBL dissemination and persistence in clinically successful clones. ^28^ In our dataset, the recurrence of similar plasmid replicon markers across multiple sequence types suggests that plasmid-associated horizontal gene transfer may contribute to the dissemination of resistance determinants in this setting. Thus, the resistance burden observed here appears to reflect not only the presence of successful lineages, but also the possible contribution of mobile genetic elements across strain backgrounds. Because this study used short-read Illumina sequencing, plasmid findings are limited to detection of replicon markers and cannot fully resolve complete plasmid structures, resistance-gene co-localization, or whether identical plasmids were shared across isolates.

Beyond resistance, the widespread detection of virulence-associated loci, including genes linked to adhesion, iron acquisition, and extraintestinal survival such as *fdeC* and *ybtP/ybtQ*, underscores the clinical relevance of these colonizing isolates. The co-occurrence of antimicrobial resistance and virulence-associated determinants raises concern that the orthopaedic ward patient population may be colonized by strains with both enhanced pathogenic potential and reduced susceptibility to commonly used antibiotics. This is especially important in orthopaedic settings, where prolonged hospitalization, surgical interventions, trauma-related wounds, and repeated antimicrobial exposure may increase opportunities for colonization, persistence, and progression to infection. ^11, 12^ From an infection prevention and antimicrobial stewardship perspective, colonized patients should therefore be viewed not simply as passive carriers, but as potential reservoirs of clinically important multidrug-resistant organisms that may influence empiric therapy decisions and IPC prioritization. ^21– 24^

The pairwise SNP analysis adds an important epidemiological dimension to these genomic findings. Although the overall SNP distance distribution was wide, indicating substantial diversity, two distinct low-SNP clusters were identified. One cluster comprised isolates that were genomically indistinguishable at the core genome level, while the second comprised isolates differing by only 0–3 SNPs. These patterns are compatible with recent shared ancestry, possible recent transmission, or shared-source acquisition within the ward. ^46^ Importantly, however, these clusters were observed against a backdrop of many genetically unrelated isolates, indicating that low-SNP relatedness is only part of the overall genomic picture. This mixed scenario suggests that ESBL-producing *E. coli* in the orthopaedic ward likely arise through both ongoing circulation of closely related strains and repeated introduction of unrelated lineages from colonized patients entering the hospital. Such a pattern is epidemiologically plausible in a setting where patient turnover, referral pathways, and prior healthcare exposure may introduce new resistant strains into the ward population ( Figure 1).

**Figure 2.**
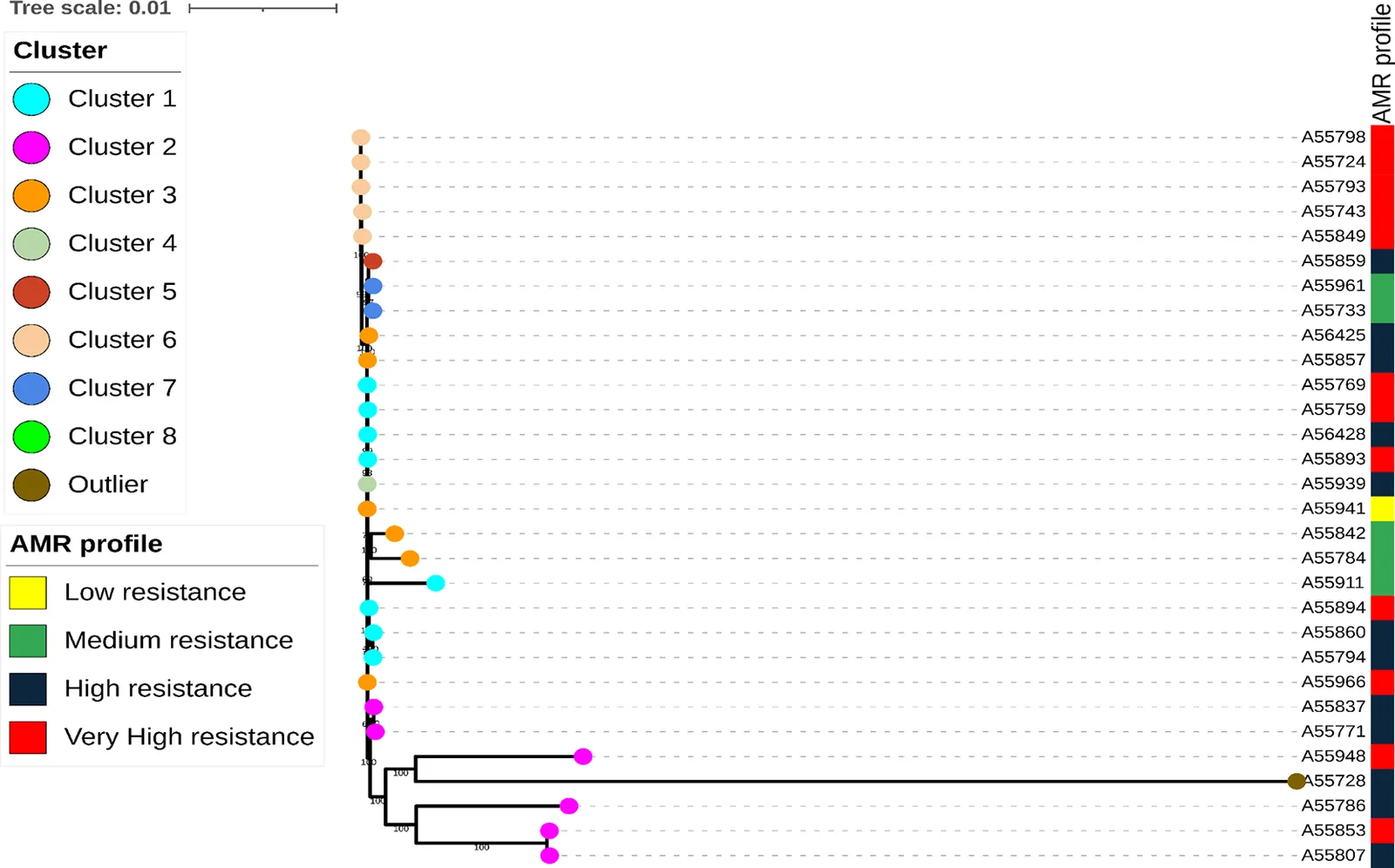
Core genome phylogenetic relationships, antimicrobial resistance profiles, and phylogroup distribution of ESBL-producing *E. coli* isolates. Core genome SNP-based phylogenetic tree of ESBL-producing *E. coli* isolates recovered from stool or rectal swabs of patients admitted to an orthopaedic ward in Mwanza, Tanzania. The tree was constructed from a recombination-filtered core genome alignment generated using Snippy, with recombination regions identified and masked using Gubbins, and maximum-likelihood inference performed using IQ-TREE. Isolates are grouped into eight phylogenetic clusters (Clusters 1–8) and one genetically distinct outlier, indicated by colored symbols at the tips. The adjacent annotation strips show the AMR profile and phylogroup assignment for each isolate. Several clusters contain closely related isolates with short branch lengths and low pairwise SNP distances, compatible with recent shared ancestry, possible recent transmission, or shared-source exposure, whereas others show greater divergence, reflecting multiple independent lineages circulating within the ward. These phylogenetic clusters represent genomic lineages or related groups and should not be interpreted as confirmed transmission chains. Isolate-level sequence type, phylogroup, predicted serotype, putative pathotype-associated marker profile, AMR determinants, plasmid replicon markers, and virulence-associated genes are provided in Extended Data Table S1.

The recombination-filtered phylogeny and shared variant analysis support this interpretation. Several isolates formed tight clusters with short branches and shared variant profiles, compatible with recent shared ancestry, possible recent transmission, or shared-source exposure. These observations highlight the value of combining phylogenetic structure, SNP distances, shared variant patterns, and available epidemiological metadata when interpreting genomic relatedness and possible circulation patterns. In a setting such as this, reliance on one metric alone could oversimplify the epidemiology; instead, the data support a model in which possible localized circulation of selected lineages and genomic convergence through shared resistance determinants may occur simultaneously. ^16, 18, 26^

From a clinical and public health perspective, these findings highlight the importance of colonized patients as reservoirs of multidrug-resistant organisms with implications for infection prevention and antimicrobial stewardship. ^47, 48^ They reinforce the value of gastrointestinal colonization surveillance in hospitalized patients, particularly in high-risk wards such as orthopaedics, where colonized patients may serve as reservoirs for onward spread and possible subsequent infection. ^11, 14^ Second, they support the need for strengthened infection prevention and control measures aimed at reducing opportunities for onward spread while also recognizing the role of patient importation of resistant strains. ^25^ Third, they highlight the utility of genomic surveillance for distinguishing closely related low-SNP clusters from broader background diversity, thereby informing targeted interventions. In resource-constrained settings, this kind of genomic information may be especially valuable for prioritizing IPC strategies and antimicrobial stewardship around the organisms and lineages most likely to be introduced, persist, or circulate locally. ^26^

This study has limitations that should be acknowledged. First, only 39 isolates passed sequencing quality control and were included in the genomic analyses, which limits the breadth of inference. Second, the study focused on patient-derived stool and rectal swab isolates and did not include contemporaneous sequencing of environmental or healthcare-worker isolates, limiting our ability to evaluate environmental reservoirs or reconstruct possible transmission pathways. Third, the analysis was conducted within a single orthopaedic ward at one tertiary hospital, and the findings may therefore not be fully generalizable to other wards, hospitals, or regions. Fourth, although plasmid replicons and resistance genes were identified, the use of short-read sequencing limits full resolution of plasmid architecture, resistance-gene co-localization, and the exact genomic context of some resistance determinants. Finally, while low SNP thresholds can identify isolates compatible with recent shared ancestry, possible recent transmission, or shared-source exposure, genomic similarity alone cannot establish the direction or exact route of transmission in the absence of denser temporal, spatial, patient-contact, and environmental sampling data. These limitations mean that our findings should be interpreted as evidence of genomic relatedness and possible circulation patterns rather than definitive proof of specific transmission events.

Despite these limitations, this study provides important genomic evidence that ESBL-producing *E. coli* colonizing orthopaedic patients in Mwanza comprise a multidrug-resistant and genomically diverse population with evidence of low-SNP relatedness among a subset of isolates. The co-circulation of high-risk international lineages, widespread *bla*_CTX-M-15_, common IncF-family plasmid replicon markers, and low-SNP clusters suggests that the orthopaedic ward may function as a point of convergence for imported resistant strains and a setting that may permit possible localized circulation. These findings underscore the need to integrate genomic surveillance into infection prevention and antimicrobial resistance monitoring frameworks in sub-Saharan African hospitals, particularly in wards where prolonged stays and antibiotic exposure may amplify the risks of colonization, persistence, and possible onward spread. ^6, 26^

## Conclusions

This study demonstrates that ESBL-producing *E. coli* colonizing patients in an orthopaedic ward in Mwanza, Tanzania comprise a multidrug-resistant, genomically diverse population dominated by *bla*_CTX-M-15_ and distributed across multiple globally recognized and locally circulating lineages. Despite this overall diversity, the identification of low-SNP clusters indicates recent shared ancestry, possible shared-source exposure, or possible localized ward-level circulation among a subset of isolates, highlighting the coexistence of multiple introductions and closely related lineages. These findings should be interpreted cautiously because genomic similarity alone cannot establish direct transmission pathways

The widespread presence of IncF-family plasmid replicon markers and shared resistance determinants across phylogenetically distinct isolates suggests that mobile genetic elements, alongside the presence of successful lineages, may contribute to shaping the resistance landscape. The concurrent detection of virulence-associated genes further underscores the clinical significance of these colonizing strains as potential sources of difficult-to-treat infections.

Together, these findings emphasize the importance of gastrointestinal colonization as a reservoir for antimicrobial resistance in hospitalized patients and highlight the need for strengthened infection prevention and control measures, improved antimicrobial stewardship, and the integration of genomic surveillance into routine monitoring frameworks in resource-limited healthcare settings.

## Software availability

The bioinformatics analyses in this study were conducted using the rMAP-2.0 (Rapid Microbial Analysis Pipeline). The pipeline is openly available and can be accessed via GitHub:

• Repository: https://github.com/gmboowa/rMAP-2.0
• Version: v2.0
• License: MIT License

A representative report including sample quality control (QC) metrics and downstream analysis outputs is available at: https://gmboowa.github.io/rMAP-2.0/reports/esbl_ecoli.html

## Ethics approval and consent to participate

This study was conducted in accordance with the Declaration of Helsinki and relevant national regulations. It was approved by the Joint CUHAS/BMC Research and Ethics Committee (CREC/409/2019) and the National Health Research Ethics Review Committee of the National Institute for Medical Research (NIMR/HQ/R.8a/Vol. IX/3322) in Tanzania. Permission to conduct the study was obtained from the hospital administration, the Head of the Department of Orthopaedics, and ward supervisors/in-charge nurses prior to sample and data collection.

Sampling did not involve collection of identifiable patient information. In the parent study, written informed consent (or assent for children) was obtained from all participants where clinical samples and data were collected. Patients with suspected surgical site infections received appropriate clinical management, including culture and antimicrobial susceptibility testing to guide therapy.

## Data Availability

All sequencing data generated in this study have been deposited in the National Center for Biotechnology Information under BioProject accession PRJNA1452460. Raw sequencing reads for all *E. coli* isolates are available in the Sequence Read Archive and are linked to this BioProject. The corresponding BioSample accession numbers are SAMN57232750–SAMN57232788, and these are also listed in the Extended data 1 (Table S1) associated with this study. Bioinformatics analyses were performed using reproducible workflows implemented in rMAP-2.0 (Rapid Microbial Analysis Pipeline). The scripts and workflow used for data processing and analysis are available at https://github.com/gmboowa/rMAP-2.0. The following Extended dataset is provided:
•
**Extended data 1 (Table S1)**: Metadata for all isolates, including BioSample accessions, sample information, sequencing and assembly quality metrics, sequence type, phylogroup, predicted serotype, putative pathotype-associated marker profile, AMR determinants, plasmid replicon markers, virulence-associated genes, and associated genomic characteristics. This dataset is available on Figshare: https://doi.org/10.6084/m9.figshare.32016402 Internal isolate identifiers used in the manuscript are not included in the public dataset to ensure de-identification; corresponding SRA accession numbers are provided in Extended data Table S1. All data are openly available without restriction in accordance with F1000Research open data policies.
