# Supplementary material for: Genomic characterization of ESBL-producing Escherichia coli isolates from patients in an orthopaedic ward in Mwanza, Tanzania
Source: F1000Res. 2026 Jun 25;15:670. Originally published 2026 May 5. [Version 2] doi: 10.12688/f1000research.180294.2 (PMC13712626; doi:10.12688/f1000research.180294.2)
Supplement: Peer review history

# Reviewer report: Reviewer response for version 2

**DOI:** 10.5256/f1000research.203890.r511362

**Published:** 2026-08-21

**Authors:** Benard Kulohoma

**Affiliations:** 1 Ortholog, Nairobi, Kenya

The study provides a valuable contribution towards understanding transmission of E. coli in resource limited settings. It is a well thought out and presented study. 

**Minor point: **I request the authors to include in the discussion that "short-read assembly fragmentation makes it challenging to  complete plasmid closure, while studying resistance genes; and this can be revisited with long-read sequencing studies in the future”

Is the work clearly and accurately presented and does it cite the current literature?

Yes

If applicable, is the statistical analysis and its interpretation appropriate?

Yes

Are all the source data underlying the results available to ensure full reproducibility?

Yes

Is the study design appropriate and is the work technically sound?

Yes

Are the conclusions drawn adequately supported by the results?

Yes

Are sufficient details of methods and analysis provided to allow replication by others?

Yes

Reviewer Expertise:

Genomics, Bioinformatics, genetics, microbial genomics, infectious diseases, data sceince, math modellling

I confirm that I have read this submission and believe that I have an appropriate level of expertise to confirm that it is of an acceptable scientific standard.

# Reviewer report: Reviewer response for version 2

**DOI:** 10.5256/f1000research.203890.r496562

**Published:** 2026-07-01

**Authors:** Sijo Asokan

**Affiliations:** 1 Mar Athanasios College for Advanced Studies Tiruvalla (MACFAST- Autonomous), Kerala, India

The author has addressed all revisions, the manuscript can be accepted for indexing.

Is the work clearly and accurately presented and does it cite the current literature?

Partly

If applicable, is the statistical analysis and its interpretation appropriate?

Partly

Are all the source data underlying the results available to ensure full reproducibility?

Yes

Is the study design appropriate and is the work technically sound?

Yes

Are the conclusions drawn adequately supported by the results?

Yes

Are sufficient details of methods and analysis provided to allow replication by others?

Yes

Reviewer Expertise:

Medical Microbiology and Infectious Diseases

I confirm that I have read this submission and believe that I have an appropriate level of expertise to confirm that it is of an acceptable scientific standard.

# Reviewer report: Reviewer response for version 1

**DOI:** 10.5256/f1000research.198895.r490132

**Published:** 2026-06-29

**Authors:** Christopher H. Connor

**Affiliations:** 1 University of Melbourne, Melbourne, Australia

Mboowa et al. present their analysis of a collection of ESBL-producing E. coli collected from an orthopaedic ward in Tanzania. Their collection of 39 isolates was whole genome sequenced and analysed for AMR gene carriage, phylogenetic relatedness and inferred transmission events. They identified common globally dispersed sequence types and AMR genes. They also report several clusters of genetically highly related strains which they suggest are the result of transmission events. Their work makes an important contribution to surveillance of a critical pathogen in an under-surveyed region. The paper is well written, and the methods are clearly described, however I have serious concerns about their chosen methodology (detailed below). Consequently, some of their interpretations may be speculative. Furthermore, the result sections are vaguely worded, repetitive, and with little to no quantification provided. 

 MAJOR COMMENTS

 Not enough information is provided on their quality control processes and why certain isolates were excluded. They state that several metrics were checked at various points throughout the analysis, for example DNA concentration, quality, purity, number of contigs, assembly N50 etc., but there is no information on what thresholds they used to decide if a sample passed or failed. Why were 3 independent species identification methods used? Were there discordant results? A total of 6 isolates were discarded, why? When the authors report metrics on genome size and number of contigs, did these metrics include the dropped samples? If not, why was there an included sample with over 3000 contigs?

 Recombination identification and removal was performed across the full dataset. Identification of recombination events is typically performed on a dataset of closely related genomes, for E. coli this is usually within a single sequence type, or even within a subclade of that sequence type. I am not confident that Gubbins could reliably identify recombination at the E. coli species scale given the high degree of genomic variability within the species. I would recommend the authors drop this analysis and refer them to a publication which has systematically investigated recombination masking [1]. The authors should check and report the size of their core genome alignment produced with Snippy, alongside the number of variant sites in the alignment. Given the diversity of STs in their dataset, I suspect the core genome alignment will only be a fraction of the E. coli genome. They should also check that Snippy isn’t imputing the non-variant sites from the reference genome. They should also check how many variant sites are being masked by recombination filtering. For the transmission inference analysis, I would instead recommend pairwise snippy mapping between de novo genome assemblies, or a reference free approach such as split k-mer analysis. Correcting this analysis could fundamentally alter their detection of transmission events and subsequent interpretation.

 The results reported are vaguely worded and no quantification is provided. For example, the authors state that there was a ‘wide range of variant sites’, ‘the SNP distribution showed heterogeneity’ and ‘some isolates showing minimal divergence’. Statements like this should be supported with quantification, or a figure. Additionally, the authors should avoid the use of the word ‘approximately’, please report the exact number rounded appropriately. Furthermore, the authors have not reported how many sequence types were identified in total, how many ST131 isolates were there? Were there any novel sequence types? What was the definition for a dominant ST? How many AMR genes were identified? How many isolates had blaCTX-M-15? In which sequence types? Did they find any carbapenem resistance genes? What quinolone resistance point mutations did they find? How many isolates had virulence genes? Which genes did they have? Which ST had the most? How many plasmid replicons were identified? In how many isolates? The authors need more quantification and supporting figures throughout their results section. Lastly, many of the results sections are highly repetitive of each other. For example, it is repeatedly stated that ‘the dataset showed evidence of recent transmission alongside genetically diverse strains’. The section on comparative analysis repeats results that have already been reported. Could the authors please report results once in the relevant section and avoid repetition.

 In figure 2 there is no description of how the authors defined a phylogenetic cluster, nor how they defined their AMR profile in figure 2. Why are some clusters spread across the tree (cluster 1 and 3)? What method for clustering did they use, single linkage?

 The methods state that epidemiological data was collected but there are no results for that data. Were their identified transmission events supported with patient data? Were patients in proximity to each other? Were they admitted within the same time frame?

 From their data provided in supplementary table 1, it looks like they are counting point mutations as resistance genes which is incorrect (e.g. mutations in gyrAB and parCE). Point mutations should be analysed and reported separately. They are also including efflux genes such as acrF and mdtM, while these genes can be associated with resistance, they are intrinsic and their connection to an AMR phenotype is complex. Furthermore, the authors have included blaEC genes which again are intrinsic and of limited clinical importance, typically they are filtered out from analyses. The data table also reports many of their isolates having a resistance gene class against fosfomycin, this is surprising as the prevalence of fosfomycin resistance in E. coli is globally low and the mechanisms are not well understood. Could the authors investigate which genes are reporting a resistance to fosfomycin? And confirm that these are genuine resistance mechanisms? I would recommend the use of the tool AMRFinderPlus and abriTAMR, the latter can filter out intrinsic resistance genes and more accurately places AMR genes into their resistance classes. Again, adjusting this analysis may fundamentally alter their results and interpretation.

 MINOR COMMENTS

 While the methods are detailed and reasonably clear I was confused by the tool rMAP. Providing their analysis pipeline is of great benefit to the field and aids reproducibility. However, this paper would benefit from a restructuring of the methods section to more clearly introduce rMAP at the start, then provide more specific details later. I was confused that rMAP was first mentioned in the Genome characterization and typing section with little explanation, then mentioned again in the AMR section with more detail. I would suggest condensing into a single section with something along the lines of “AMR, plasmid, virulence gene detection, typing and characterization were performed with rMAP (link and citation). AMR genes in rMAP are detected with ResFinder… etc.”

 Are the authors able to provide more information on the incidence of ESBL positive isolates? How many samples were screened? How many were ESBL negative? This information would be beneficial for global AMR surveillance efforts.

 Could the authors provide more context on their patients, specifically were these patients admitted via emergency departments? Or transferred from elsewhere in the hospital network? What was their prior exposure to clinical environments where they may have been susceptible to colonisation with ESBL E. coli. Are patients in this hospital routinely screened for ESBL E. coli?

 What CLSI breakpoint versions were used for EBSL identification? Are the authors able to report the disc diffusion zones and compare that with their genomic data?

 The authors haven’t detailed what method was used for DNA extraction, a kit of some sort?

 The authors are using a bespoke library preparation protocol (LITE), has this protocol been published? Can they provide a citation? 

 Were the mlst and snippy tools run with default parameters, none were specified.

 Was snippy-core used to generate the core genome alignment? As above it is also important to report the size of the resulting alignment and number of variant sites.

 Results table 1 blends patient with bacterial isolate characteristics, can these be more clearly distinguished or separated please.

Is the work clearly and accurately presented and does it cite the current literature?

Partly

If applicable, is the statistical analysis and its interpretation appropriate?

Not applicable

Are all the source data underlying the results available to ensure full reproducibility?

Yes

Is the study design appropriate and is the work technically sound?

Partly

Are the conclusions drawn adequately supported by the results?

No

Are sufficient details of methods and analysis provided to allow replication by others?

Partly

Reviewer Expertise:

Microbial genomics, E. coli population genomics, antimicrobial resistance, pathogen transmission

I confirm that I have read this submission and believe that I have an appropriate level of expertise to state that I do not consider it to be of an acceptable scientific standard, for reasons outlined above.

# Reviewer report: Reviewer response for version 1

**DOI:** 10.5256/f1000research.198895.r490131

**Published:** 2026-06-11

**Authors:** Sijo Asokan

**Affiliations:** 1 Mar Athanasios College for Advanced Studies Tiruvalla (MACFAST- Autonomous), Kerala, India

- The authors should clearly explain why only 39 of the 45 isolates passed quality control and were included in the final analysis. Specific reasons for exclusion should be provided.
- Table 1 indicates that isolates originated from both patients and the environment, whereas the methods primarily describe patient stool and rectal swab samples. The source of environmental isolates and their sampling strategy should be clarified.
- The manuscript would benefit from inclusion of sequencing quality metrics such as average coverage depth, read counts, assembly completeness, and N50 values for individual isolates or as supplementary material.
- The rationale for selecting a ≤5 SNP threshold to infer transmission should be discussed in greater detail, including references supporting its applicability to ESBL producing *E. coli* in this epidemiological context.
- Although transmission clusters were identified, integration of temporal and spatial patient data would strengthen the evidence for recent transmission events. The authors should discuss this limitation more explicitly.
- The results section would be strengthened by providing the exact prevalence of major sequence types, resistance genes, plasmid replicons, and virulence determinants rather than descriptive statements alone.
- Additional details regarding the distribution of blaCTX-M-15 among different sequence types would improve understanding of lineage specific dissemination patterns.
- The authors report widespread IncF plasmids but do not provide detailed plasmid characterization. A discussion of the limitations of short read sequencing for plasmid reconstruction should be expanded.
- Figure 2 presents phylogenetic clusters, but the biological significance of the eight clusters should be described more clearly in the text.
- Inclusion of antimicrobial resistance gene frequencies in a supplementary table would improve data accessibility and reproducibility.
- The discussion appropriately links the findings to global dissemination of high risk clones such as ST131 and ST1193. However, additional comparison with similar genomic studies from other African healthcare settings would strengthen the regional relevance of the findings.
- The manuscript would benefit from a brief discussion regarding the potential clinical implications of colonization by these multidrug resistant strains, including progression to infection and impact on antimicrobial stewardship programs.
- The limitations section is appropriate but should additionally acknowledge the relatively small sample size and the absence of long read sequencing for complete plasmid resolution.
- The conclusions are supported by the presented genomic data and are consistent with the results obtained. However, statements regarding transmission should remain cautious because genomic similarity alone cannot establish direct transmission pathways.
- Cite these references properly in the discussion portion:
- refer 1,2,3

Is the work clearly and accurately presented and does it cite the current literature?

Partly

If applicable, is the statistical analysis and its interpretation appropriate?

Partly

Are all the source data underlying the results available to ensure full reproducibility?

Yes

Is the study design appropriate and is the work technically sound?

Yes

Are the conclusions drawn adequately supported by the results?

Yes

Are sufficient details of methods and analysis provided to allow replication by others?

Yes

Reviewer Expertise:

Medical Microbiology and Infectious Diseases

I confirm that I have read this submission and believe that I have an appropriate level of expertise to confirm that it is of an acceptable scientific standard, however I have significant reservations, as outlined above.

## References

[1] Asokan S, Banerjee N, Saleem M, Atiyah HM, Pandey RK, Abbas RK, Yousif SI, Radhamanalan G, Parashar A, Gowtham B, Balaji VK. Healthcare associated infections (HAI): insights into epidemiology, microbiology, and diagnostics. Diagnostic Microbiology and Infectious Disease. 2026 Mar 12:117376.10.1016/j.diagmicrobio.2026.11737641865593

[2] Asokan S, Pandey RK, Jalil MA, Abd Alhussen SK, Yousif SI, Abbas RK, Vijayan S, Rajeswary D, Jacob T, Atiyah MM. Biofilm associated infections on medical devices: pathogenesis, diagnostic challenges, and control strategies. The Microbe. 2026 Apr 3:100712.

[3] Asokan S, Choudekar A, Jagadeesan A, Sm R, Ali A, Napte SU, Selvam SA, Verma G, Das P, Beniwal N, Radhamanalan G. Molecular diagnostics in clinical microbiology: Advances, applications, and future directions. Diagnostic Microbiology and Infectious Disease. 2025 Dec 10:117223.10.1016/j.diagmicrobio.2025.11722341406849

# Reviewer report: Reviewer response for version 1

**DOI:** 10.5256/f1000research.198895.r481994

**Published:** 2026-05-18

**Authors:** Ayorinde O Afolayan

**Affiliations:** 1 Leibniz Institute - German Collection of Microorganisms and Cell Cultures (DSMZ), Germany, Germany

The study investigated the genomic characteristics of ESBL-carrying *Escherichia coli* recovered from patients admitted into the orthopaedic ward in Mwanza, Tanzania over a 3-month period. The identification of strains belonging to globally recognized sequence types highlights the international dissemination of these pathogens and their associated antimicrobial resistance (AMR) genes and virulence genes. The observed transmission clusters suggests possible in-hospital (within-ward) spread among vulnerable patients. The study contributes valuable data to the limited scientific knowledge on the spread of ESBL gene-carrying *E. coli* strains in low- and middle-income countries, and points to the need for routine genomic surveillance in order to inform strategies and policies needed to reduce their spread. 

 Methodology: It will be useful to know the phylogroup, serotype, and pathotype of the *E. coli* strains (including whether any pathogen carried virulence genes associated with more than one pathotype).

 Some of the embedded web-links are non-functional (e.g., https://gmboowa.github.io/rMAP-2.0/reports/assets/sections/A55724_blast.html, returned a 'Page not found' error message)

 Concordance (or level of agreement) between genotypic AMR prediction results and phenotypic antimicrobial susceptibility testing (AST) results should be reported.

 Authors should consider adding a table summarizing the SNP ranges and mean SNP distance per sequence type.

 Under the sub-heading 'Pairwise SNP distances and transmission inference', the authors mentioned that 'a subset of isolates exhibited very low SNP distances (0-5)...'. Authors should specify how many isolates exhibited very low SNP distances, and the sequence type to which they belong.

 Table 2: It is not clear whether some of the *E. coli* strains were recovered from the same patient or not.

 Figure 2: This figure would benefit from the inclusion of relevant epidemiological data alongside the phylogenetic tree. Midpoint-rooting the tree may also improve interpretation. The figure legends should not appear pixelated.

 Readers would also benefit from a careful examination of the figure associated with the transmission network analysis,  as it provides important visual context for the inferred transmission clusters and relationships among the isolates.

 The study could be strengthened by placing the strains within the context of other *E. coli* genomes from Tanzania, the rest of Africa, and globally. This would help clarify whether the strains reflect single or multiple introductions into Tanzania, as well as potential regional or international dissemination patterns.

Is the work clearly and accurately presented and does it cite the current literature?

Yes

If applicable, is the statistical analysis and its interpretation appropriate?

Not applicable

Are all the source data underlying the results available to ensure full reproducibility?

Partly

Is the study design appropriate and is the work technically sound?

Yes

Are the conclusions drawn adequately supported by the results?

Yes

Are sufficient details of methods and analysis provided to allow replication by others?

Yes

Reviewer Expertise:

Genomic surveillance of antimicrobial resistance

I confirm that I have read this submission and believe that I have an appropriate level of expertise to confirm that it is of an acceptable scientific standard, however I have significant reservations, as outlined above.
